# Up-regulated fibronectin in 3D culture facilitates spreading of triple negative breast cancer cells on 2D through integrin β-5 and Src

**DOI:** 10.1038/s41598-019-56276-3

**Published:** 2019-12-27

**Authors:** Hwa -Jeong Park, David M. Helfman

**Affiliations:** Department of Biological Sciences, Korea Advanced Institute of Science and Technology, Daejeon, Korea

**Keywords:** Breast cancer, Extracellular matrix

## Abstract

Using MDA-MB-231 cells as a model of triple negative breast cancer (TNBC) and its metastatic sub-cell lines that preferentially metastasize to lung, bone or brain, we found that the mRNA and protein levels of fibronectin (FN) are increased in MDA-MB-231 cells and its lung metastatic derivative, when cultivated in three-dimensional (3D) suspension cultures. The increase of FN expression in 3D was dependent on p38 mitogen-activated protein kinase (MAPK) because it was prevented by treatment of cells with SB203580, an inhibitor of p38MAPK. The up-regulated FN was converted into fibrils, and it enhanced cell spreading when cells cultured in 3D were transferred to two-dimensional (2D) culture. The arginine-glycine-aspartate (RGD) peptides and siRNAs targeting of integrin β-5 inhibited spreading of cells regardless of the presence of FN on 2D culture dishes. In addition, the levels of phosphorylated Src were found to be increased in 3D and the treatment of cells with SU6656, an inhibitor of Src, decreased the rate of cell spreading on FN. Collectively, these studies demonstrate that increased cellular FN in 3D suspension culture facilitates cancer cell attachment and spreading via integrin β-5 and Src, suggesting that the increased FN promotes initial attachment of cancer cells to secondary organs after circulation during metastasis.

## Introduction

Although conventional 2D culture systems have provided many insights into cell biology and therapeutic treatment of cancer, the development of new culture systems to better mimic *in vivo* situations provides additional insights into cancer cell behavior. Comprehensive and systematic studies have illuminated distinctively different gene expression and signaling cascades profiles between cells cultured in 2D and in 3D cell culture systems and it is thought that 3D culture better reflects the physiological behavior of cells^1–4^.

Cells grown in 3D culture exhibit adaptive characteristics to the environment, different from those of cells grown in 2D culture. When cells are cultured on 2D surfaces, cells display large focal adhesions in which more than 100 different proteins including integrins can assemble and communicate bi-directionally with extracellular matrix (ECM)^5^. Thus, cells adhered on 2D surfaces induce intracellular signaling through focal adhesions. In addition, signals from inside cells can determine migration speed, persistence, and directionality by influences on focal adhesion dynamics. In contrast to cells cultured in 2D, cells grown in 3D soft matrix possess smaller focal adhesions that diffuse not only in the basal part, but also across the surface of the cells^6,7^. To efficiently negotiate in 3D conditions, the cell using protrusive dynamic rather than regulating the size of focal adhesion binds to, moves on, and releases the accessible ECM fibrils surrounding the cell.

As cancer progression develops, tumor cells undergo metastasis which consists of multiple steps including invasion through tissues via penetration of the basement membrane, intravasation to the circulatory system to move through the blood or lymph, and extravasation from the circulation system, followed by colonization in the second organ as a new niche^8^. During this process, tumor cells in the circulatory system inevitably remain detached from the scaffolding structures of tissue. The environment of the circulatory system is unfavorable for circulating tumor cells (CTCs) to be viable and to initiate metastasis, since the CTCs can be attacked by immune cells and Reactive Oxygen Species, and large focal adhesions providing appropriate survival signal are absent in them^9^. Nonetheless, some cancer cells survive in the vascular system and successfully metastasize to secondary organs.

Triple negative breast cancer is an aggressive subtype of breast cancer characterized by lack of expression of estrogen receptor (ER), progesterone receptor (PR), and human epidermal growth factor receptor (HER2) and accounts for more than 10% of all breast cancers^10,11^. Because the majority of TNBC cells do not possess a specific target, it is relatively difficult to find an effectively available treatment, and generally has an adverse prognosis with a high risk of recurrence and metastasis and resistance to conventional therapy. MDA-MB-231 cells, a model TNBC cell line, were injected into immunodeficient mice, and the cells showing organ-specific metastasis to lung, bone, or brain were classified^12,13^. Through the *in vivo* study of microarray and functional genomics, a number of genes mediating lung metastasis of MDA-MB-231 cells were identified.

In the present study, we used 2D and 3D culture systems to study cellular behaviors that might facilitate metastasis. We identified that FN is highly up-regulated in MDA-MB-231 (herein referred to as parental) and its lung metastatic derivative (herein referred to as LM2), but not in bone and brain metastatic derivatives, when they are specifically cultured in 3D suspension condition. Considering that FN, which is a marker of epithelial-mesenchymal transition (EMT) and a crucial component of ECM, is not expressed in normal adult breast tissues and its expression is up-regulated during breast cancer development^14,15^, we investigated the role of increased cellular FN in 3D suspension cultures when cells encounter 2D surfaces. We found that increased cellular expression of FN in 3D conditions facilitates cancer cell attachment and spreading via integrin β-5 and Src, suggesting that increased FN promotes initial attachment of cancer cells to secondary organ after circulation during metastasis.

## Results

### Fibronectin expression is increased in MDA-MB-231 and MDA-MB-231 LM2 in 3D suspension culture

**Figure 1:**
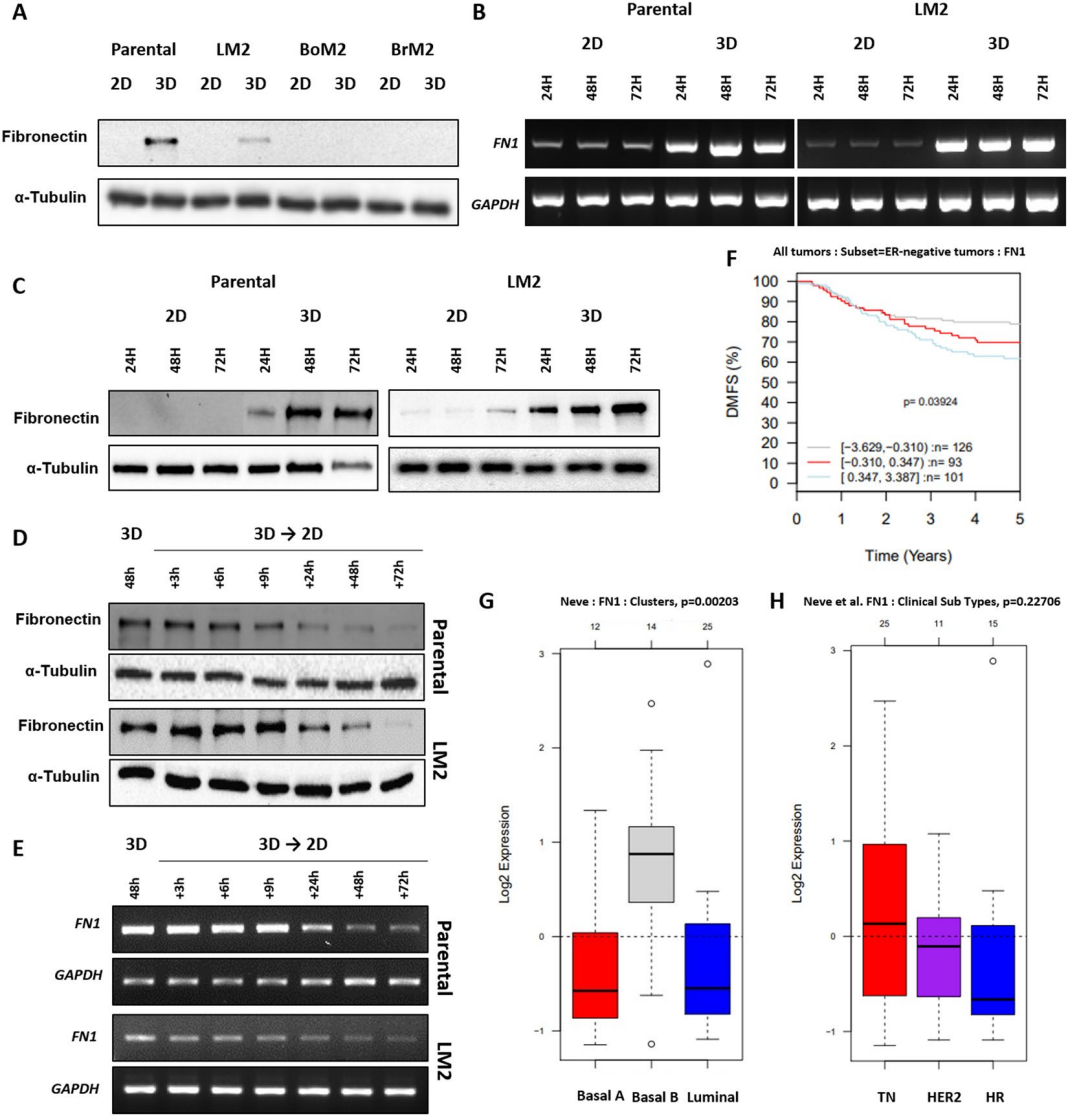
Changes in fibronectin expression of TNBCs in 3D and *in vivo* gene set analysis. (**A**) MDA-MB-231 parental, LM2, BoM2, and BrM2 were cultured in 2D or 3D condition for 48 hours, and the level of fibronectin protein was detected and the blots were cropped from different parts of the same gel. The mRNA levels (**B**) and protein levels (**C**) were monitored at 24, 48, and 72 hour in MDA-MB-231 parental and LM2 cells grown in 2D or 3D condition using electrophoretic gels and immunoblots, respectively. And the bands of blots were cropped from the same gel for each cell line (parental and LM2). MDA-MB-231 parental and LM2 cells cultured in 3D suspension conditions were transferred in 2D culture plates, and changes in fibronectin protein (**D**) and mRNA (**E**) expression were detected and the PCR products were observed using electrophoretic gels. (**F)** Kaplan-Meier survival analysis showing the relationship between *FN1* gene expression and 5-year distant metastasis free survival outcomes in patients with ER-negative breast cancer. (**G**) A box plot exhibiting variation of *FN1* gene expression according to breast cancer subtypes; basal-A, basal-B and luminal. (**H**) A box plot of *FN1* gene expression in different breast cancer cell lines using GOBO gene-set analysis. TN; triple negative breast cancer cell lines, HER2; HER2-positive breast cancer cell lines, and HR; Hormone receptor positive breast cancer cell lines.

To investigate changes induced by different culture conditions, a TNBC cell line, MDA-MB-231 cells (herein referred to as parental) and its derivatives that prefer to metastasize to lung (LM2), bone (BoM2), or brain (BrM2) were cultured in 3D ultra-low attachment plates or 2D plates for 48 hours. Interestingly, in 3D suspension culture, all four cell lines spontaneously formed floating cell spheroids that did not die, despite losing the contact surface. To identify changed expressions in EMT marker proteins between cells cultured in 2D versus 3D conditions, western blot analysis was performed using lysates of cells grown under each condition (Fig. S1A). Whereas parental and LM2 cells exhibited increases of FN expression in 3D suspension cultures, BoM2 and BrM2 did not show detectable changes in FN expression (Fig. 1A). In addition, MCF7, a non-metastatic breast tumor cell line, were examined for the change in FN expression according to culture condition. And the results showed that there was not a significant difference of fibronectin between MCF7 cells cultured in 2D and in 3D (Fig. S1B).

To determine if the increased FN proteins of parental and LM2 cells were from transcription of *FN1* gene, we conducted polymerase chain reaction with messenger RNAs (mRNAs) extracted from the two kinds of cells and it was found that both cells produced markedly increased amounts of mRNAs as well as proteins in a time-dependent manner (Fig. 1B,C). Additionally, in order to determine if the increase in the expression changes of FN is reversible, we transferred parental and LM2 cells to 2D culture dishes after incubating them in 3D for 48 hours. In both cell lines, the amounts of proteins and mRNAs of FN gradually decreased with the passage of time (Fig. 1D,E). Taken together, we concluded that FN expression in parental and LM2 cells is up-regulated only when the cells are cultured in 3D suspension condition.

### The relation between fibronectin and breast cancer based on gene-set analysis

With the results of up-regulation of FN in 3D culture, we analyzed the expression of FN in different breast cancer subtypes taking advantages of GOBO online tool (http://co.bmc.lu.se/gobo/)^16^. Interestingly, 101 cases displaying highly expressed FN in tumor tissue show a decreased pattern of distant metastasis free 5-years survival (DMSF) rate among 320 ER-negative breast cancer samples (Fig. 1F). When the data is analyzed depending on cell types, it revealed that a significantly higher expression of FN is identified in the basal B cancer cell group^17^, which is a group containing TNBC and that TNBC cell lines showed higher mean of FN expression compared to HER2-positive and/or hormone receptor-positive cell lines (Fig. 1G,H). The Cancer Genome Atlas (TCGA) breast cancer dataset was additionally analyzed, using UCSC cancer genomic browser (http://genome-cancer.ucsc.edu/)^18^ (Fig. S2). We identified that about 120 samples were classified as TNBCs among 1247 breast cancer tissues samples, and approximately 15~20% of them exhibited high expression of FN even though its expression pattern is not generally distinguished as a specific characteristic.

These results from two different data analysis tools based on Gene Expression Omnibus public repositories and TCGA dataset, respectively, are complementary for the result of our *in vitro* experiments that only parental and LM2 cells showed highly up-regulated FN in 3D although all 4 cell lines exhibited low levels of FN expression on 2D culture dishes. Furthermore, these results might be one explanation for highly expressed FN in only some TNBC patients.

### Fibronectin expression is regulated by p38 MAP kinase in 3D

**Figure 2:**
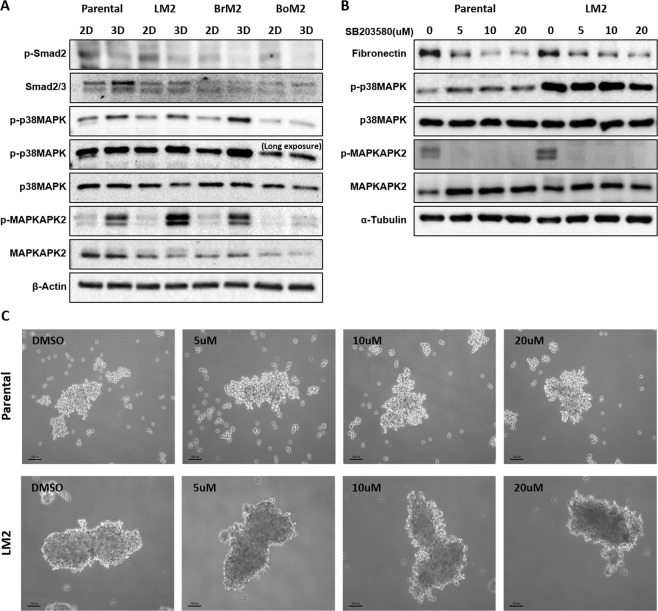
The effect of SB203580 treatment in fibronectin expression in 3D-grown cells. (**A**) Phosphorylation of p38MAPK and MAPKMAP2 increases in MDA-MB-231 parental and organotropic metastasis derivatives in 3D suspension culture for 48 hours. The same volume of the set of cell lysates were loaded into different SDS-page gels. The blots of phosphorylated MAPKAPK and p38MAPK were cropped from the same gel and the other blots were cropped from different gels. (**B**) SB203580 was added as serial concentrations in 3D culture media and changes in fibronectin protein expression were monitored after cells were cultivated for 48 hours in 3D. The same volume of the set of cell lysates was loaded into different gels and p-p38 MAPK and MAPKAPK were cropped from the same gel. The other blots were cropped from different gels. (**C**) MDA-MB-231 parental and LM2 cells were treated with the p38 MAPK inhibitor, SB203580 for 48 hours and formation of cell-spheroids was observed. Scale bar: 100 μm.

It was reported in a previous study that FN in MDA-MB-231 cells is increased by transforming growth factor-β (TGF-β) signaling via p38MAPK pathway, and p38MAPK is involved in the regulation of gene transcription and translation^19,20^. Based on this information, we investigated the role of p38 MAPK on FN expression in 3D suspension culture. Although TGF-β signaling is not active, it was found that the phosphorylation level of p38MAPK was increased in 3D suspension culture (Fig. 2A). Also, MAP kinase-activated protein kinase 2 (MAPKAPK2), a downstream protein regulated by p38 MAPK showed increased phosphorylation when cells were cultured in 3D suspension condition^21^ (Fig. 2A). To identify the influence of p38 MAPK on FN synthesis, MDA-MB-231 parental and LM2 cells cultured in 3D were treated with the p38 MAPK inhibitor, SB203580. Decreased expression of FN in both cell lines was observed in a dose-dependent manner in cells treated with SB203580, while the formation of cell spheroids was hardly affected by SB203580 at 10 μM (Figs. 2B,C and S3).

### The increased availability of fibronectin in 3D suspension culture facilitates fibrillogenesis and cell spreading when cells attach on 2D surface

**Figure 3:**
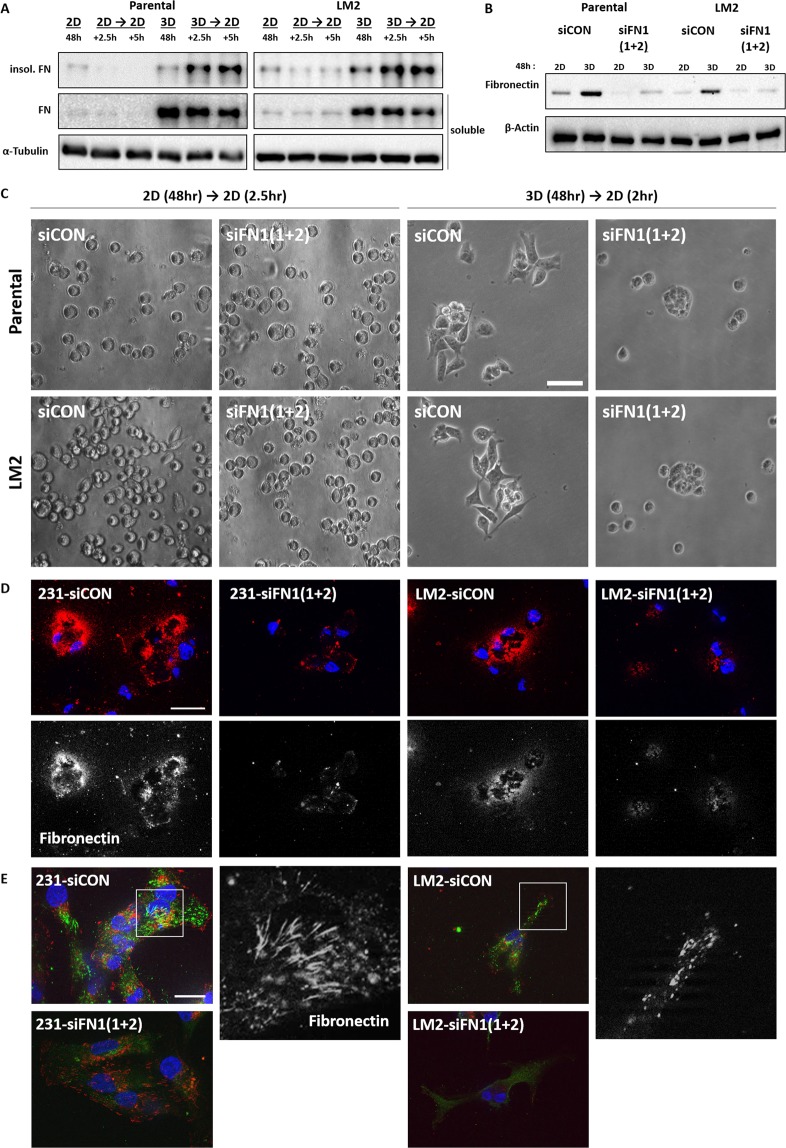
Increased fibronectin of MDA-MB-231 parental and LM2 cells in 3D facilitates cell attachment on 2D. Cells grown in 2D or 3D suspension cultures for 48 hours were re-cultivated in serum-free culture media on 2D culture plates and subjected to immunoblot after being harvested at 2.5 hr and 5 hr from 2D re-cultures and DOC-insoluble and DOC-soluble fibronectin was detected (**A**). DOC-soluble fibronectin used as internal loading control for comparison. DOC-soluble and DOC-insoluble lysates were loaded into different gels and cropped for immunoblots. (**B**) Protein expression changes were observed after *FN1* siRNAs were treated in MDA-MA-231 parental and LM2 cells. The mixture of two different kinds of siRNAs against *FN1* were transfected into cells in a serial manner in which 25 nM siRNAs were treated each time, and totally 50 nM siRNAs were treated. The blots for fibronectin and α-tubulin were cropped from the same gel. (**C**) Cells cultured in 2D or 3D condition for 48 hr were re-cultivated in 2D culture plates with serum-free media, and the spreading patterns of cells in each condition were observed. Scale bar: 50 μm. (**D**) *FN1* or control siRNA treated cells were re-cultivated on 2D culture glasses for 2.5 hours after 3D incubation for 48 hours and fixed with 4% formalin. Fixed cells were not permeabilized before antibody staining to detect only the extracellular fibronectin. DAPI was used to stain nuclei. Blue: DAPI and Red: extracellular fibronectin. Scale bar: 10 μM. The below panels show extracellular fibronectin (White). (**E**) After 3D culture for 48hours,cells transfected with control or *FN1* siRNA were cultivated for 2.5 hours and fixed, and permeabilized with PBS containing 1% deocycholate. Fixed cells were treated with antibodies against FN and phopho-paxillin, indicated squares(white line) were magnified in a black and white version. Blue:DAPI, Green:FN, and Red: phspho-paxillin. Scale bar: 20 μm.

Cellular FN that is synthesized by cells and secreted into the interstitial space of tissues first exists as a soluble dimer and undergoes fibrillogenesis to be formed as ECM fibril^22–24^. Thus, we investigated whether FN present inside cells and the interstitial space of cell spheres serves as a component of ECM after being converted to fibrils when cells encounter 2D surfaces. To separate the FN assembled into high molecular weight fibrils, the cells were spread onto 2D culture dishes after incubation for 48 hours in 2D attached or 3D suspended state, and cell lysates were harvested using deoxycholate (DOC) by which fibrillated FN is insoluble^25^. While the cells transferred from 2D to 2D did not accumulate DOC-insoluble FNs after being transferred, more DOC-insoluble FNs were detected in the pellet of from-3D-to-2D samples in a time-dependent manner (Fig. 3A).

To determine the role of cellular FN in 3D culture, two different kinds of siRNAs targeting FN gene (*FN1*) were used to knock-down the mRNA of *FN1* (Fig. 3B). When comparing *FN1* siRNA transfected cells with non-targeting siRNA control group, it was observed that knocking-down of FN reduces secreted ECM FN on the surface of culture glasses (Fig. 3D). Also, in both cell lines, loss of FN led to decreased spreading of cell bodies for two hours after being transferred to 2D from 3D suspension culture (Fig. 3C).

MDA-MB-231 parental and LM2 cells moved from 3D to 2D culture glasses were immunolabled to test whether FN secreted from the cell grown in 3D was converted into the fibers. Paxillin, which is known to localize at focal adhesions was co-stained in order to analyze cell-attachment surface and to identify if FN fibers were formed as the cells adhered on the surface. In accordance with the increase of DOC-insoluble FN, it appeared that the FN fibers were formed by both of the cell lines, although LM2 cells showed smaller FN fibers than MDA-MB-231 parental cells (Fig. 3E).

### Increased fibronectin in 3D facilitates adhesion on 2D surfaces and a HUVEC monolayer

**Figure 4:**
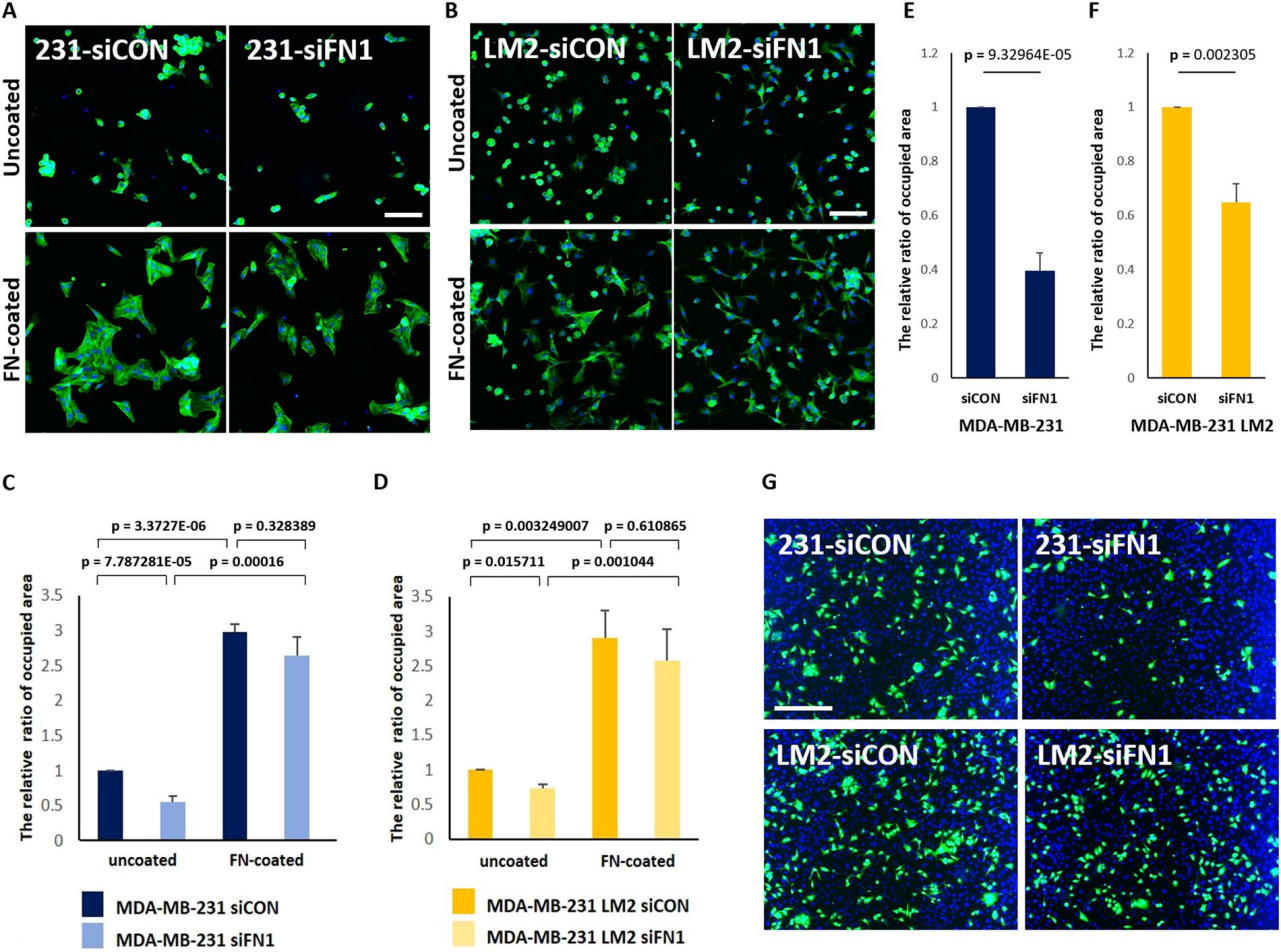
Down-regulation of fibronectin in 3D suspension culture reduces attachment of MDA-MB-231 parental and LM2 cells to HUVECs. (**A,B**) MDA-MB-231 parental and LM2 cells were re-cultivated for 2.5 hours on uncoated or fibronectin VI-coated culture glasses, fixed with 4% formalin and stained with phalloidin (green) and DAPI (blue) and (**C,D**) the areas covered with cells that were attached to 2D culture glasses were measured. Scale bar: 100 μm. (**G**) MDA-MB-231 parental and LM2 cells were re-plated on a HUVEC monolayer for 2 hours, after being cultured in 3D suspension condition for 48 hours. Breast cancer cells were stained with green CMFDA, and the nuclei of breast cancer cells and HUVECs were stained DAPI (blue). Scale bar: 200 μm. (**E,F**) The relative ratio of attached MDA-MB-231 parental and LM2 cells on HUVECs was measured, based on CMFDA. For the experiments the mixture of two kinds of *FN1* siRNA were transfected to parental and LM2 cells. (means ± s.e.m; Student’s *t*-test).

To determine if the different cell spreading patterns are due to the presence of FN fibers, the cells grown in 3D were spread on uncoated coverslips or coverslips coated with FN-IV. In all four cell lines, increased rate of cell adhesion to 2D were observed on FN-coated culture glasses elucidating that fibronectin is effective promoter for attachment of cells grown in 3D (Figs. 4A–D, S4A–C). Also, the relative areas covered with the transferred cells were higher in control group than in *FN1* siRNA-transfected group on uncoated culture glasses, whereas differences in attachment rates was not significant on FN-IV-coated plates (Fig. 4A–D). Therefore, these results suggest that the cells cultivated in 3D suspension condition that have high availability of FN facilitates binding of cells to attachable surfaces by being converted into ECM fibrils.

Metastasis is a step-wise process including extravasation during which cancer cells are required to escape from the circulatory system to secondary organs through efficient adhesion and penetration of a vascular vessel. We investigated the influence of the 3D-specific increase of FN on cell adhesion to human umbilical vein endodermal cells (HUVECs) since we hypothesized that enhanced FN availability of cancer cells in 3D contributes to metastasis by increasing cell adhesion to a second niche. MDA-MB-231 parental cells and LM2 cells were transferred from 3D culture on pre-cultured HUVEC monolayer. Depletion of FN reduced the cell adhesion rate onto HUVECs for 2 hours in both cell lines (Fig. 4E–G). Thus, this result suggests that the increase of FN in tumor cells has a role in cell adhesion on blood vessels during metastasis.

### Increase of fibronectin in 3D facilitates cell-attachment from 3D to 2D via collaboration with integrin β-5

**Figure 5:**
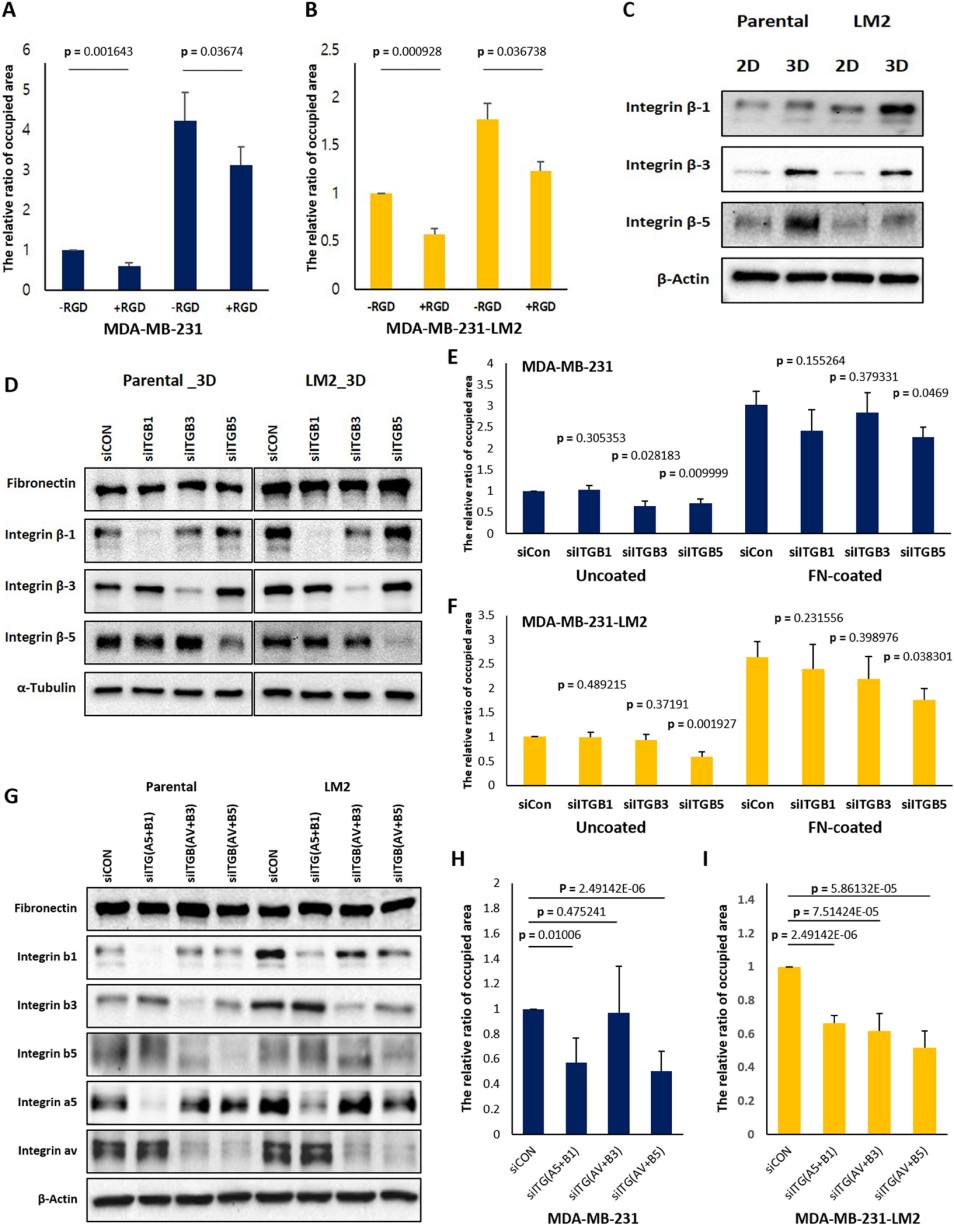
RGD peptides and integrin β-5 silencing disturb cell attachment from 3D to 2D. (**A,B**) 500 μM RGD peptides were added into 2D serum-free culture media before cells grown in 3D were transferred on uncoated- or fibronectin coated-2D culture glasses and the cells attached on 2D surfaces were fixed after 2.5 hours. And the areas occupied by the attached cells were measured. (**C**) Changes in protein levels of each integrin β isoform in different culture conditions were detected, and (**D**) the protein levels of fibronectin, integrin β-1, integrin β-3, and integrin β-5 of the respective siRNAs treated cells were examined by western blot. The same volume of the set of cell lysates were loaded in different gels to conduct immunoblots for each integrin β isoforms, fibronectin, β-actin, and α-tubulin and the blots were cropped from the gels. (**E,F**) The siRNA transfected cells were transferred to 2D culture plates uncoated or coated with fibronectin after 3D incubation for 48 hours and the areas covered by attached cells were measured. The mixtures of two different kinds of siRNAs against ITGB1 and ITGB5 and a siRNA against ITGB3 were used for siRNA transfection, respectively. Cells were transfected with siRNAs at concentration of 25 nM in a serial manner, total 50 nM. (**G**) siRNAs targeting different integrin isoforms were transfected to parental and LM2 cells in three different combinations, and 25 nM siRNA were treated per gene; ITGA5, ITGAV, ITGB1, ITGB3, and ITGB5 (totally 50 nM siRNA for a combination). The mixture of two different kinds of siRNA for ITGA5 and ITGAV were used. (H and I) siRNA transfected cells with each combination were spread on fibronectin coated culture glasses for 2.5 hours after being cultured in 3D suspension condition. And the areas covered by adherent cells were compared with those of control siRNA treated cells. (means ± s.e.m; Student’s *t*-test).

FN monomers contain many interaction sites for different types of integrin heterodimers^26^. The RGD motif, the triple amino acids (Arg-Gly-Asp) sequence present within many ECM proteins, was originally identified as being located within a FN molecule^27^. This small peptide found in FN interacts effectively with various integrin heterodimers including α5β1, αvβ3 and αvβ5^28,29^$.$ To examine whether the differences in cell attachment were due to the RGD region of FN, cells harvested from 3D plate were incubated in media containing RGD peptides prior to being transferred to 2D. Reduced attachment of cells was observed by the treatment of RGD peptides, showing that RGD peptides effectively interfere with the binding process of cells (Fig. 5A,B).

We next examined the effects of three different integrin β isoforms (integrin β-1, β-3, and β-5) using corresponding siRNAs because it has been reported that integrin αvβ3, α5β1, and α5β1 display the most, second and third affinity values to RGD peptides, respectively^28^. In addition, we found that these three integrin β isoforms were increased in MDA-MB-231 parental and LM2 cells cultured in 3D (Fig. 5C). Depletion of integrin β-5 deceased attachment of the cells grown in 3D onto FN-coated culture glasses, suggesting that the increased FN in 3D facilitates cell adhesions via interaction with integrin β-5 (Fig. 5E,F).

Additionally, we did double knocking-down of each type of integrin heterodimers; α5β1, αvβ3 and αvβ5, using siRNAs to identify whether αvβ5 heterodimers are crucial for cell adhesions on FN (Fig. 5G). Silencing of αvβ5 heterodimers caused significant reduction of the adhesion on FN in parental cells, and downregulation of three different integrin heterodimers influenced on the adhesion rate of LM2 cells (Fig. 5H,I). Remarkably, downregulation of integrin αvβ5 showed the most reduction of cell adhesion on FN-coated culture glasses in both cell lines.

### Src family kinase is required for cell attachment from 3D to 2D

**Figure 6:**
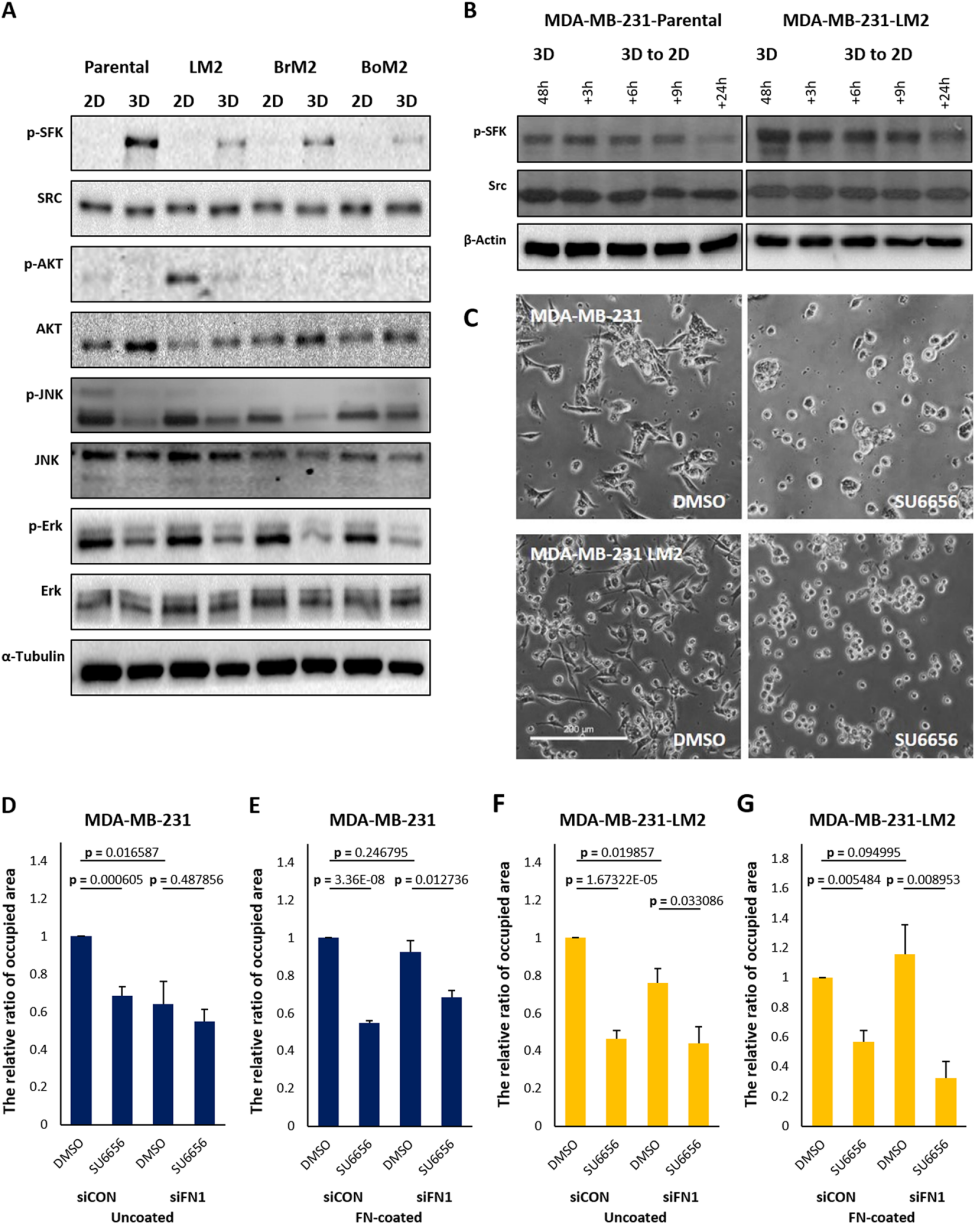
Pharmacological inhibition of SFK reduces the rate of cell-attachment from 3D to 2D. (**A**) Cells grown in 2D or 3D cultures for 48 hours were harvested and subjected to immunoblot analysis and phosphorylation of signaling proteins were detected. The same volumes of the set of cell lysates were loaded in different gels to carry out immunoblots for p-SFK, Src, Erk, JNK, AKT and α-tubulin, and the blots were cropped from different gels. (**B**) The changed levels of phosphorylated SFK were observed by western blot. The cells were transferred to 2D culture plate containing serum-free media after 3D suspension culture for 48 hours, and the cell lysates were harvested according to the order of time. The same volume of the set of cell lysates were loaded in different gel to carry out immunoblots for p-SFK, Src and β-actin. (**C**) SU6656 was added to culture media 30 min before transferring cells from 3D to 2D and the patterns of cell spreading were observed after 2.5 hours. Scale bar: 200 μm. (**D–G**) Control or fibroenctin down-regulated cells were re-cultivated on uncoated- or fibronectinVI coated-culture glasses after being cultured in 3D suspension condition. Before transferring the cells to 2D, 10 μM of SU6656 was added. The areas covered by attached cells were measured and compared. (means ± s.e.m; Student’s *t*-test).

Cells cultured in 3D environments exhibit different architecture and signaling networks compared with cells grown in 2D. MDA-MB-231 parental and the organ-specific metastatic derivatives also exhibit changes in phosphorylation levels of signaling proteins in 3D compared to 2D. While PI3K/AKT pathway, JNK pathway, TGF-β pathway, and Ras-Erk pathway were down-regulated, Src family kinase (SFK) showed higher phosphorylation levels in 3D suspension culture (Fig. 2A and 6A). The levels of phosphorylated SFK gradually decreased when cells were re-cultivated in 2D following 3D suspension culture for 48 hours (Fig. 6B).

SFK is a key regulator for focal adhesion formation^30^, and it was reported that the loss of function mutant in Src disrupted proper focal adhesion formation and migratory behavior of cells. We investigated the role of Src using the SFK inhibitor, SU6656, on 2D-attachment of cells which had been cultured in 3D. The treatment of cells with SU6656 inhibited cell spreading on 2D surface, leaving the cells round in shape without noticeable adherent structures (Fig. 6C). In addition, the rate of cell attachment was also reduced by SU6656 on uncoated or FN-coated culture glasses, except for parental cells treated with FN siRNA on uncoated condition, suggesting that Src activity is more crucial for attachment of parental cells to FN fibrils (Fig. 6D–G). Thus, it appears that the activity of SFKs are required for cells to spread and form protrusions on 2D surfaces when they are transferred from 3D even though there are large amounts of FN in cell spheroids.

### Down-regulated fibronectin of MDA-MB-231 parental cells causes decreased collective migration of cell-monolayers derived from 3D cellular aggregates

**Figure 7:**
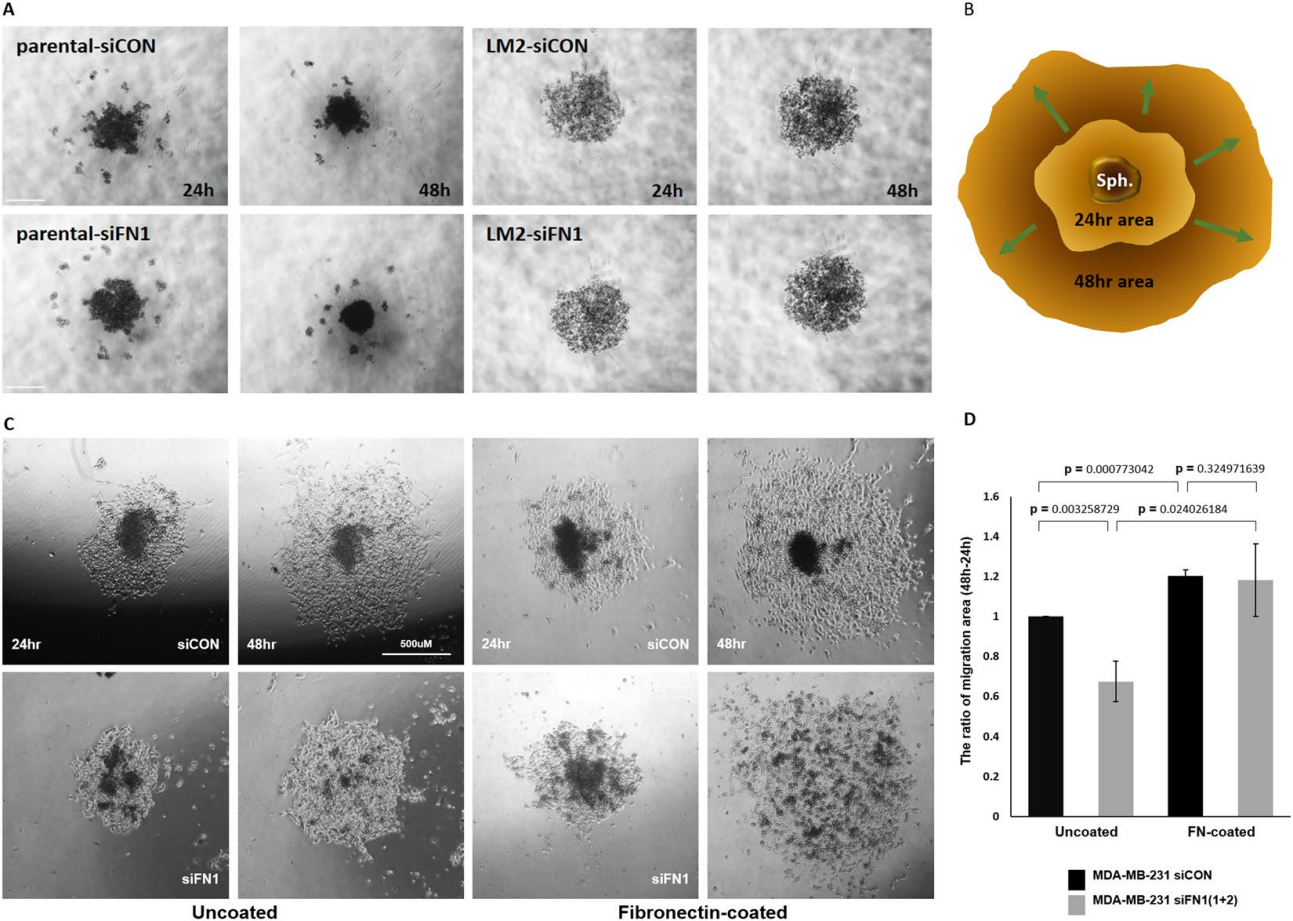
Downregulation of fibronectin induces retarded collective migration of cellular monolayer derived from 3D cell-aggregates. (**A**) *FN1* or control siRNA transfected MDA-MB-231 parental and LM2 cells (5,000 cells/well) were cultured in ultra-low attachment 96-well round bottom plates to form aggregates for 48 hours. Scale bar: 500 μm. (**C,D**) MDA-MB-231 parental cell-aggregates were transferred to uncoated or fibronectin VI-coated culture plates and the areas covered by cellular monolayer derived from the aggregates were measured by using ImageJ. The covered area with cellular monolayer at 24 hour was deducted from that at 48 hour. (**B**) A cartoon explains migration of cellular monolayer derived from cell-aggregates. (means ± s.e.m; Student’s *t*-test).

To further investigate the influence of increased FN on cell migration when cells were moved from 3D to 2D attachable surfaces, the cellular aggregates formed in ultra-low attachment round bottom plates were transferred to 2D culture-plates (Figs. 7 and S5). LM2 cells and BrM2 cells were excluded from the aggregate transferring experiment because they did not form compact cellular aggregates and they dispersed when being transferred. Also, it is worth noting that LM2 cells form only few multicellular tumor spheroids through comparative study on spheroid formation^31^. After being transferred from ultra-low attachment round bottom plates, parental or BoM2 cellular monolayers on 2D plates were derived from cell aggregates, and the area of cell migration at 24 hour was subtracted from the area of cell migration at 48 hour, and the net migration area for 24 hours were measured and compared (Fig. 7B). As expected, coating the plate with FN facilitated parental and BoM2 cell migration on 2D surfaces from the aggregates formed in 3D (Figs. 7C and S6A,B), confirming a crucial role of FN. Given that BoM2 did not display the increase of FN in 3D condition, depletion of FN using siRNAs was applied to only parental cells and reduced migration from the 3D cell aggregates was observed (Fig. 7C,D). This reduction was rescued when the parental spheroids were transferred onto FN-coated culture plate. Taken together, these results lead to the conclusion that increased FN in 3D suspension condition promotes MDA-MB-231 cell migration on 2D.

## Discussion

Recently, the use of conventional 2D culture systems has been replaced by advanced culture systems that better mimic the *in vivo* environment. Using 3D culture systems, it is possible to gain new insights to bring a better understanding of diverse biological phenomena, such as in development and tumor progression. Here, we undertook a study using 3D culture conditions in a TNBC cell line and identified a potential role of FN in breast cancer metastasis. We observed that 3D suspension culture conditions resulted in the increase of FN expression, activation of different signaling pathways and cellular behaviors associated with metastasis.

We discovered that the mRNA and protein of FN were increased when cells are cultured in 3D with MDA-MB-231 parental and its lung specific metastatic sub-line, LM2 (Fig. 1). Although it has previously been shown that FN is increased in 3D suspension culture in studies using fibroblasts, which is a mesenchymal cell, our experimental results showed that the TNBC cells derived from epithelium do not express FN under normal 2D conditions, but they do when cultured in 3D^32^. Results of a systemic analysis of data on human breast cancer patient samples support a role of FN in breast cancer progression. Based on two different data sets, it was confirmed that the mean value of FN expression of TNBCs is higher, compared to HER2 positive or hormone receptor positive breast cancers. Also, FN overexpression is correlated with a poor 5-year survival rate in patients with TNBCs. MDA-MB-231 and three organotropic metastasis sublines used in the present study exhibit low expression of FN in 2D culture. By contrast, parental and LM2 cells showed up-regulated FN expression in 3D suspension culture. Interestingly, only 15 to 20% of TNBC patients display overexpression of *FN1* mRNA (Fig. S1). Taken together, our observation of the changes in FN expression determined by culture system might be one possible explanation for this phenomenon related to *FN1* expression in TNBC patients.

Cells grown in 3D suspension condition showed a gradual decrease in FN when cells were transferred to 2D (Fig. 1). Interestingly, we could not find specific data on the relationship between FN expression and metastasis to specific organs through analysis of databases. It is possible that their FN expression underwent a change through Mesenchymal-Epithelial Transition (MET) during the colonization at new sites because human breast cancer samples were acquired from tumor attached to primary or metastatic sites as a solid form^33^. Thus, data obtained from patient samples and our *in vitro* experimental results may not contradict each other. In this respect, our experimental result is reminiscent of observation that mesenchymal positive CTC clusters which are tagged by probes for *FN1* transcripts account for much more portion than epithelial positive CTC clusters from a study using human blood samples^34^. It was also reported that a junction protein called plakoglobin plays an important role in oligo-CTC cluster formation, oligo-CTC clusters showed high propensity to metastasize to lung than single CTCs in mice, and that the stability of *FN1* mRNA is regulated by plakoglobin^15,35,36^. Also, tenascin C, an ECM protein known to interact with FN enhances invasiveness and outgrowth of lung micrometastases of MDA-MB-231-LM2 cells in mice^15,37–39^. Therefore, two observations that MDA-MB-231 parental and lung metastatic MDA-MB-231 cells spontaneously form spheroids by themselves and that FN is up-regulated in 3D suggest FN plays an important role during the circulation of tumor cells.

Although it was found that FN increased in 3D in MDA-MB-231 parental and LM2 cells, E-cadherin was also up-regulated in 3D. One possible explanation is cancer cells might survive in the circulation system using strengthened cell-cell interaction through E-cadherin and this interaction could promote collective migration on attachable surfaces following exit from the circulation. Thus, the role of increased E-cadherin in 3D should be further studied.

We found that FN expression is dependent on the activity of increased p38 MAPK in 3D because the treatment of cells with SB203580 inhibited the expression of FN and activity of MAPKAPK (Fig. 2). This is in agreement with the previous research demonstrating that p38 MAPK activity mediated by TGF-β increases FN expression in MDA-MB-231 cells^19^. However, different from previous studies, our experimental results showed that the 3D culture environment itself could increase the expression of FN without additional stimuli such as TGB- β to activate p38 MAPK. Since 3D suspension environment does not provide appropriate survival signals such as focal adhesions on 2D, it appears that cells in 3D activate p38MAPK by different mechanisms to increase adaptability that still wait to be elucidated.

Silencing of endogenous *FN1* expression using siRNAs reduced the proportion of cells adhering and spreading to the culture glass and HUVEC monolayer in both parental and LM2 cells, suggesting that the increased expression of FN in 3D provides a favorable environment for cells to quickly and efficiently bind to blood vessels (Fig. 4). In addition, we found that this attachment is also mediated through integrin ${\rm{\beta }}$-5 (Fig. 5). However, it is still necessary to further investigate if integrin β-5 is involved in either binding to FN fibrils or fibrillogenesis. There are two possibilities to explain the role of integrin ${\rm{\beta }}$-5. First, integrin ${\rm{\beta }}$-5 only functions as an interacting partner to FN fibrils because reduced cell spreading by integrin ${\rm{\beta }}$-5 silencing was not rescued on FN coated plates. Another possibility is that integrin β-5 is also involved in the conversion of soluble FN to ECM fibrils because cells transferred from 3D suspension culture showed a decrease in the rate of spreading on uncoated-culture glasses in the case of integrin β-5 silencing. This reduced binding to uncoated-culture glasses might be due to failure to assemble FN fibrils or due to reduced binding to the formed FN fibrils.

We found that the phosphorylation levels of intracellular signaling proteins Smad, AKT, ERK, and JNK are reduced, but SFK is increased in 3D. We also showed that the activity of SFK is necessary for cells to adhere to FN fibrils (Fig. 6). Given that Src plays an important role in the regulation of size and dynamics of focal adhesions of migrating cells and that enhanced activation and genetic mutation of Src promote oncogenesis^30,40–42^, there is a possibility that up-regulated FN, SFK, and integrins contribute not only to survival in the 3D environment in the circulation but also to the adaptation when cells come in contact with new sites as metastatic organs.

Efforts have been made to clarify the characteristics of metastatic cells showing a tendency to metastasize to specific organs. However, there are data for and against the ‘soil and seed’ hypothesis, because all other metastatic organs, other than the primary source, do not already provide a hospitable environment for metastatic cancer cells. Alternatively, the ‘seed bringing their own soil’ hypothesis is that the circulating metastatic cells deliver the factors necessary for their own adaptation to survive at the new niche as disseminated tumor cells (DTCs) and the evidence supporting this has gradually accumulated^43^. It was reported that CTC clusters traveling in the body vessels have better metastatic efficiency than single CTCs since CTC clusters can take diverse strategies for successful metastasis, such as protecting themselves from immune cell’s attacks and containing necessary survival factors such as TGF-β and PDGF in CTC clusters^44^. And our results showed that MDA-MB-231 parental and LM2 cells are able to generate their own structurally supportive components by themselves and to maintain up-regulated integrin signaling for dissemination, although our experimental 3D culture condition has limitations to exactly mimic *in vivo* microemboli containing stromal cells, cytokines, and other additive components as well as tumor cells. Thus, this could be supportive evidence for the latter hypothesis i.e., the seed bringing its own soil. It was demonstrated that DTCs derived from breast cancer cell secrete collagen crosslinking enzyme LOX and PLOD2, thereby amplifying focal adhesion signaling mediated by integrin by stiffening ECM^45,46^. Considering these biological findings, the activation mechanism of genes involved in the formation of FN fibril when TNBC cells are transferred from 3D to 2D should provide further insights into the role of FN in metastasis.

## Methods

### Reagents and antibodies

Reagents. DMSO (Sigma, D8401), SB203580 (Calbiocam, 559389), SU6656 (Sigma, S9692), and RGD peptides (Sigma, A8052).

Antibodies. Fibronectin (BD Biosciences, 610077), α-tubulin(Sigma, T5168), β-actin (Sigma, A5441), integrin β-1 (Cell Signaling Technology, 4706), integrin β-3(D7X3P) (Cell Signaling Technology, 13166), integrin β-4 (Santa Cruz Biotechnology, sc-514426), integrin β-5 (Santa Cruz Biotechnology, sc-5402), integrin α-5 (Cell Signaling Technology, 4705), integrin α-v (Cell Signaling Technology, 4711) ERK1 (Santa Cruz Biotechnology, sc-94), p-ERK(T202/Y204) (Cell Signaling Technology, 9101), AKT (Cell Signaling Technology, 9272), p-AKT(S473) (Cell Signaling Technology, 9271), SAPK/JNK (Cell Signaling Technology, 9258), p-SAPK/JNK(T183/Y185) (Cell Signaling Technology, 9251), Smad2/3 (Cell Signaling Technology, 3102), Src (Cell Signaling Technology, 2110), p-Src(Y416) (Cell Signaling Technology, 2101), p38MAPK (Cell Signaling Technology, 9211), p-p38MAPK (Cell Signaling Technology, 9212), MAPKAPK2 (Cell Signaling Technology, 3042), p-MAPKAPK2(Thr334) (Cell Signaling Technology, 3007), and), p-paxillin (Y118) (Cell Signaling Technology, 2541).

### Cell culture

MDA-MB-231 parental cells, LM2 cells, BoM2 cells, and BrM2 cells were provided as a generous gift from Dr. Joan Massague. These cell lines were cultured in Dulbecco’s modified Eagle’s medium high glucose (Welgene, South Korea) supplemented with 10% fetal bovine serum (JR Scientific, Woodland, CA, USA), 100U/ml penicillin and 100 mg/ml streptomycin with L-glutamine (Welgene). For 3D culture, cells were incubated in ultra-low attachment surface, flat bottom plate (Corning). Human Umbilical Vein Cells (HUVECs) (Science cell, USA) were provided as a generous gift from Dr. Miyoung Kim. HUVECs were cultivated in Endothelial Cell Growth Basal Medium-2 (Lonza, USA) supplemented with EGM^TM^-2 MV Microvascular Endothelial SingleQuots^TM^ Kit (Lonza, USA) on 0.1% gelatin (Welgene, South Korea) coated culture plates or glasses. All cells were cultured at 37 °C in a humidified 5% CO_2_ incubator.

### Reverse-transcriptase polymerase chain reaction

Total messenger RNAs were extracted from cells using RiboEx (Geneall, South Korea) and Hybrid-R^TM^ extraction kit (Geneall, South Korea) according to manufacturer’s instruction. The concentration of total mRNA was measured by Nonodrop spectrometer (Thermoscinetific). Generation of first strand cDNA from total mRNA and amplification of this cDNA strand were reacted together using Prime Script^TM^ one step RT-PCR kit Ver2 (TAKARA, Japan) and PCR machine (Bio-rad, Richmond, USA) according to manufacturer’s instructions. Differently expressed PCR products were separated by 1.2% agarose gel electrophoresis. Specific primer sequences are as followed: FN1 fwd, 5′-AAACTGCAAACTCCGTCACC-3′; FN1 rev, 5′-AGACAGAGGGACCCAACTTG-3′ GAPDH fwd, 5′-GTCAGTGGTGGACCTGACCT-3′; GAPDH rev, 5′-AGGGGAGATTCAGTGTGGTG-3′

### Small-interfering RNA knockdown

Cells were seeded into 60 mm plate at ~30% confluent for serial transfection. After incubation for 24 hours, cells were primarily transfected with siRNAs against FN1 (Bioneer, South Korea), ITGB1 (Bioneer, South Korea), ITGB3 (Bioneer, South Korea), ITGB5 (Bioneer, South Korea), ITGA5 (Bioneer, South Korea), ITGVA (Bioneer, South Korea), or negative control siRNA (Bioneer, South Korea) using Lipofectamine RNAi Max (Invitrogen, USA) according to manufacturer’s protocols. Primarily transfected cells incubated for 48 hour at 37 °C in a humidified CO_2_ incubator at 5%. Additionally, cells were secondly transfected with siRNA using Lipofectamine RNAi Max and incubated for additional 48 hours before indicated experiments.

### Adhesion assay

Cells were collected after incubation for 48 hours in a 3D culture plate by centrifugation at 300 × g for 5 minutes. After used culture media were removed by aspiration, cells were resuspended with serum-free DMEM and agitated for 30 minutes in an incubator at 37 °C. 1 × 10^5^ Cells were replated on 2D culture glasses in 24-well plate and incubated for 2 and a half hours at 37 °C in a humidified CO_2_ incubator at 5%. To coat the glasses with fibronectin (0.001%), fibronectin-IV was purchased from Sigma (Sigma, F0895). After unattached cells were removed by aspiration, cells on culture glasses were co-fixed and permeabilized with 0.1% Triton-X-100 (Sigma-Aldrich, USA) in 4% formaldehyde (Sigma-Aldrich, USA) for 10 minutes, and stained with phalloidin conjugated with Oregon green (Invitrogen, USA) for 30 min at RT. 4,6-Diamidino-2-pheylindile (DAPI) (1 μg/ml) was incubated for 5 minutes. Prolong gold antifade reagent (Invitrogen, USA) was used for mounting coverslips. Cell fluorescence was observed with ZEISS Observer Z1 microscope with Apotome 2. Image acquisition and processing were performed with AxioVision 4.8. Cell attached area was measured by ZEISS AxioVision4.8AutoMeasure module (Zeiss, Germany).

For cell adhesion assay on HUVEC monolayer, 5 × 10^4^ HUVECs were seeded and cultured on gelatin coated culture glasses for 48 hours. MDA-MB-231 parental and LM2 cells were stained with CellTracker ^TM^green 5-Chloromethylfluorescein diacetate (CMFDA) (Invitrogen, USA) in serum-free media for 30 minutes. Resuspended MDA-MB-231 parental cells or LM2 cells were re-plated on HUVEC monolayer and cultured for 2 hours at 37 °C in a humidified CO_2_ incubator at 5% and followed by fixation with 4% formaldehyde. Fixed co-cultivated cells were stained with DAPI.

### DOC fraction

Deoxycholate fraction of fibronectin was conducted according to the previously published protocol^25^. Briefly, cells attached on 2D surface or collected from 3D culture plate were washed with ice-cold PBS, followed by being lysed with DOC buffer (2% Deoxycholate, 0.02 M Tris-HCl,pH 8.8, 2 mM PMSF, 2 mM NaF, 2 mM Na7VO4, phosphatase inhibitor cocktail (Roche), protease inhibitor cocktail (Sigma)). Lysis was carried out by passing the lysate through a 23-gauge syringe needle more than ten times, followed by incubation on rotor wheel for 30 minutes at 4 °C. The lysates were centrifuged at 13,000 × g for 30 min at 4 °C, the supernatants were transferred to other tubes as the soluble fraction. Pellets were washed briefly with DOC buffer and the used buffer was discarded, remaining pellet was designated as the insoluble fraction. The insoluble fraction was resuspended by 2× laemmli sample buffer (0.25 M Tis-HCl, 20% glycerol, 4% Sodium dodecyl sulfate, 0.004% Bromophenol blue, phosphatase inhibitor cocktail (Roche), protease inhibitor cocktail (sigma)). The soluble fraction was mixed with 2× laemmli sample buffer and used for the internal comparison of ratio among samples.

### 3D cellular aggregates formation and aggregates migration assay

Cells were cultured in 96-well ultra-low attachment round bottom plates (Corning) at concentration of 5,000cells/well. The plates containing cells were centrifuged at 1000 × g for 10 min to form aggregates. The aggregates were allowed to grow in serum containing media for 48 hours at 37 °C in a humidified CO_2_ incubator at 5%. Then the cell-aggregates were transferred to culture plates or fibronectin coated culture plates in which serum containing media is present and the aggregates were cultured for 48 hours for analysis.

### Immunoblotting

Whole cell lysates were extracted by laemmli 2× sample buffer containing a cocktail of phosphatase inhibitor, PhosStop (Roche) and protease inhibitors. The total lysate of proteins separated by sodium dodecyl sulfate polyacrylamide gel electrophoresis and transferred to 0.2 μm nitrocellulose membranes for immunoblotting, the nitrocellulose membrane was incubated with primary antibodies overnight at 4 °C. Peroxidase-conjugated anti-mouse IgG and goat-rabbit IgG (Jackson ImmunoResearch), and bovine anti-goat IgG-HRP (Santa Cruz Biothechnology) were used as secondary antibodies. To visualize the proteins, peroxidase substrate (Thermo Scientific) was applied for enhanced chemiluminescence. Chemi-DOC MP image system (Bio-Rad, USA) was used to capture image.

### Immunofluorescence

Cells were collected by centrifugation (Gyrogen 1236MG) for 5 minutes at 300 × g from 3D culture plates after 48 hr incubation. Collected cells were incubated for 30 minutes in a shaking incubator at 100 rpm at 37 °C and re-plated on 2D culture plate. Cells were fixed with 4% formaldehyde (Sigma-Aldrich, USA) for 10 minutes followed by permeabilization with 1% DOC for 10 minutes or not, and incubated with primary antibodies for 30 minutes, followed by incubation with secondary antibodies for 30 minutes at RT. DAPI (1 μg/ml) was incubated for 5 minutes. Prolong gold antifade reagent (Invitrogen, USA) was used for mounting coverslips. Cell fluorescence was observed with ZEISS Observer Z1 microscope with Apotome 2. Image acquisition and processing were performed with AxioVision 4.8.

### Data mining

GOBO (http://co.bmc.lu.se/gobo/) and UCSC (http://genome-cancer.ucsc.edu/) cancer genomic browser online tools were used to evaluate the expression level of *FN1* in different subtypes of breast cancer.

### Statistical analysis

Data were expressed three or more individual experiments. For all parametric data, an unpaired two-tailed Student’s *t*-test was used to evaluate difference between groups.

## Supplementary information

Supplementary information

## Data Availability

All data generated or analyzed during this study are included in this published article (and its supplementary files).
