# Supplementary material for: Up-regulated fibronectin in 3D culture facilitates spreading of triple negative breast cancer cells on 2D through integrin β-5 and Src
Source: Sci Rep. 2019 Dec 27;9:19950. doi: 10.1038/s41598-019-56276-3 (PMC6934487; doi:10.1038/s41598-019-56276-3)
Supplement: Supplementary file 1 — Supplementary information [file 41598_2019_56276_MOESM1_ESM.pdf]

Supplementary information

Up-regulated fibronectin in 3D culture facilitates spreading of triple negative breast cancer cells on 2D through integrin β-5 and Src

Hwa-Jeong Park[1] and David M. Helfman[1*]

[1]Department of Biological Sciences, Korea Advanced Institute of Science and Technology Daejeon, Korea

*Corresponding author: David M. Helfman

Mailing address: Department of Biological Sciences, KAIST 291 Daehak-ro, Yuseong-gu, Daejeon, Republic of Korea, Telephone: 82-42-350-2687, Fax: 82-42-350-5647, E-mail address: dhelfman@kaist.ac.kr

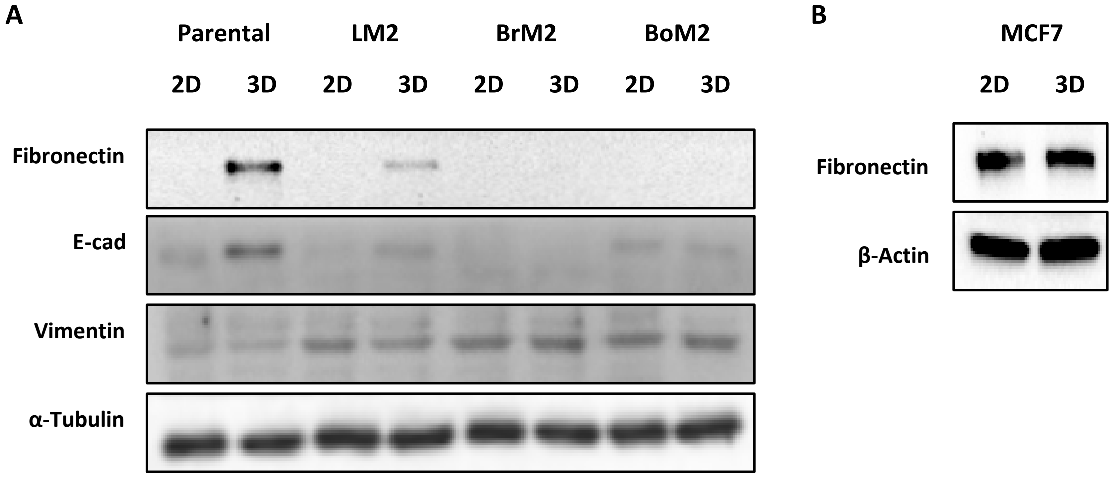

Supplementary Figure 1. The changes in EMT marker proteins in 2D or 3D suspension culture

(A) MDA-MB-231 parental, LM2, BrM2, and BoM2 were cultured on 2D or in 3D suspension culture for 48hours and the changes of fibronectin, E-cadherin, and vimentin were detected by immunoblot. The same volume of the set of cell lysates were loaded in different gels. The blots of fibronectin and α-tubulin were cropped from the same gel and the blots of E-cadherin and vimentin were cropped from the same gel. (B) 1.0 X 10^5cells/ml MCF7 cells were cultivated in 2D or 3D suspension culture and fibronectin proteins were stained with antidodies against fibronectin.

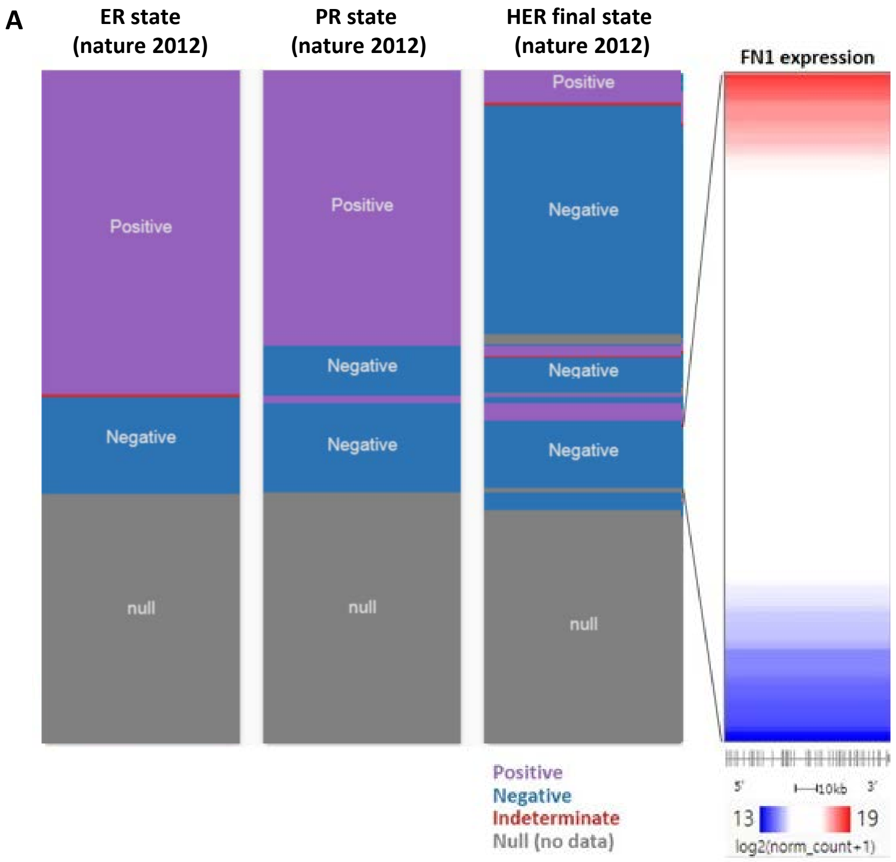

Supplementary Figure 2. Fibronectin expression variation in patients with TNBC

Fibronectin expression variation in patients with TNBC in TCGA breast carcinoma database using UCSC cancer genomic browser (http://genome.ucsc.edu/, the release date of the genome assembly:11-10-2019). To classify the type of TCGA breast carcinoma, visualization tool of UCSC xenabrowser was used (http://genome-cancer:ucsc.edu/). Among 1247 breast cancer tissues sample, about 120 samples were classified as TNBCs and approximately 15~20% of them exhibited high expression of fibronectin even though its expression pattern is not generally distinctive as a specific character. ; Purple: receptor-positive, Red: receptor-intermediate, and blue: receptor-negative.

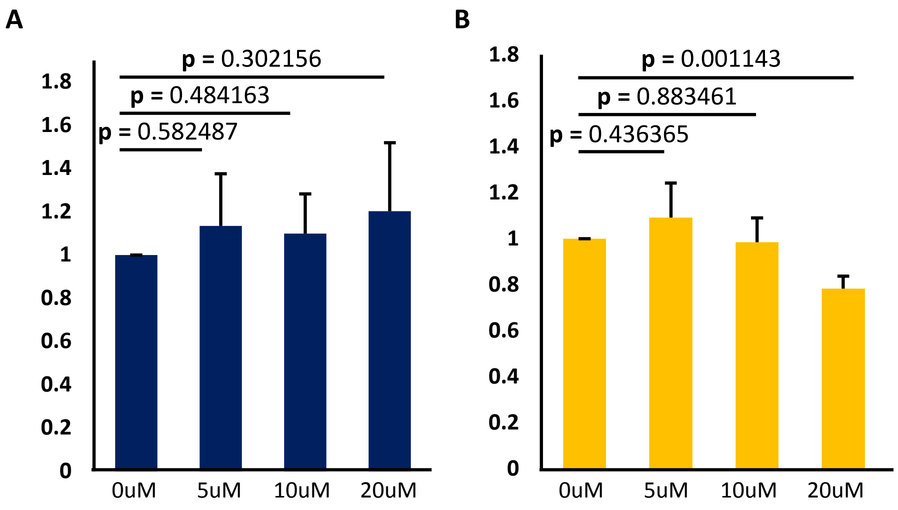

Supplementary Figure 3. The effect of SB203580 on the relative size of cell spheroids

SB203580 was added to MDA-MB-231 parental cells (A) and LM2 cells (B) in the indicated dose dependent manner in 3D suspension culture for 48hours, and the size of cell spheroids were measured by using Image J. (means ± s.e.m, Student's t-test)

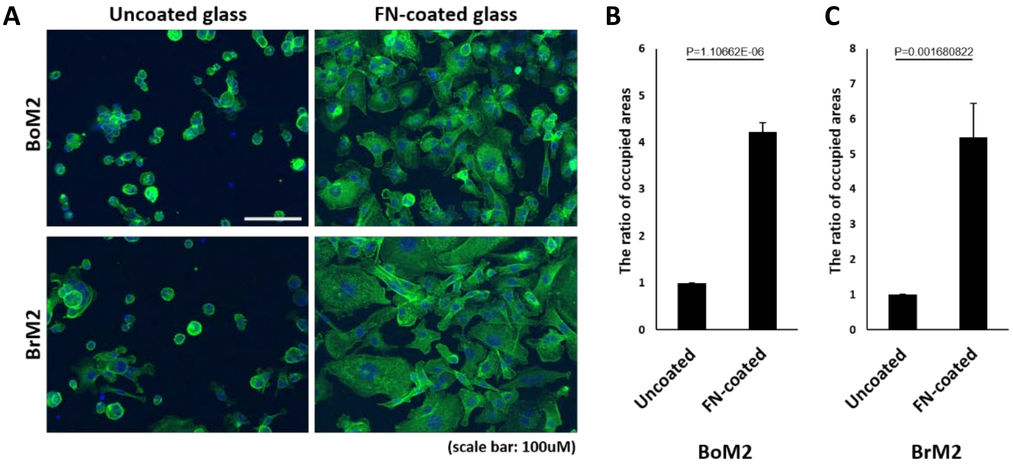

Supplementary Figure 4. The influence of fibronectin on spreading of BoM2 and BrM2 cells cultured in 3D

BoM2 and BrM2 cells grown in 3D for 48hours were re-cultivated for 2.5 hours on uncoated or fibronectin Ⅵ-coated culture glasses, fixed with 4% formalin, and stained with phalloidin (green) and DAPI (blue) (A), the areas covered with relocated cells were measured and compared by using Image J (B and C). (means ± s.e.m, Student's t-test)

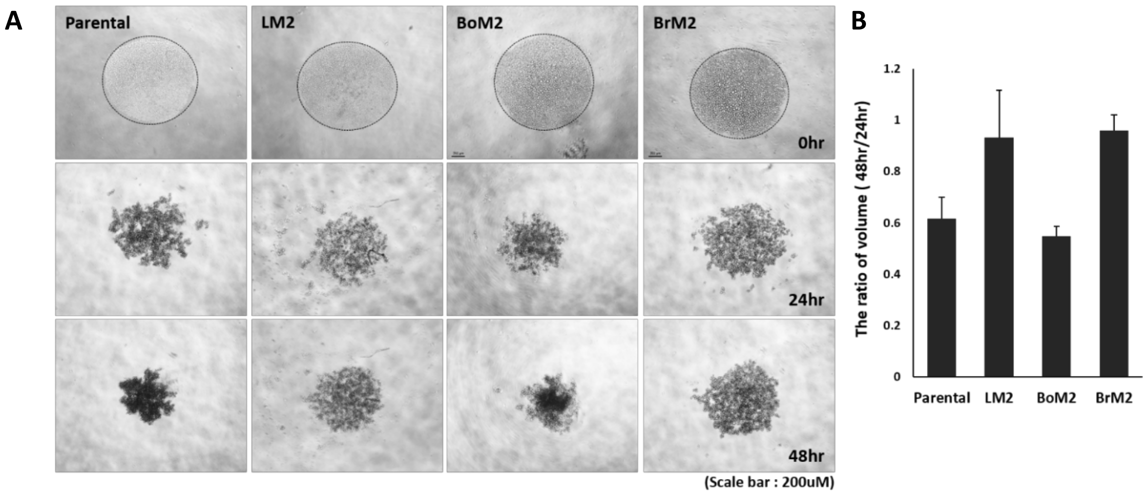

Supplementary Figure 5. The variation of cell aggregates among MDA-MB-231 parental and its organotropic derivatives

(A, B) MDA-MB-231 parental, LM2, BoM2, and BrM2 cells were cultured in ultra-low attachment 96-well round bottom plates for 48 hours and the size of cell aggregates formed in the plates were measure and compared by using Image J. (means ± s.e.m, Student’s t-test)

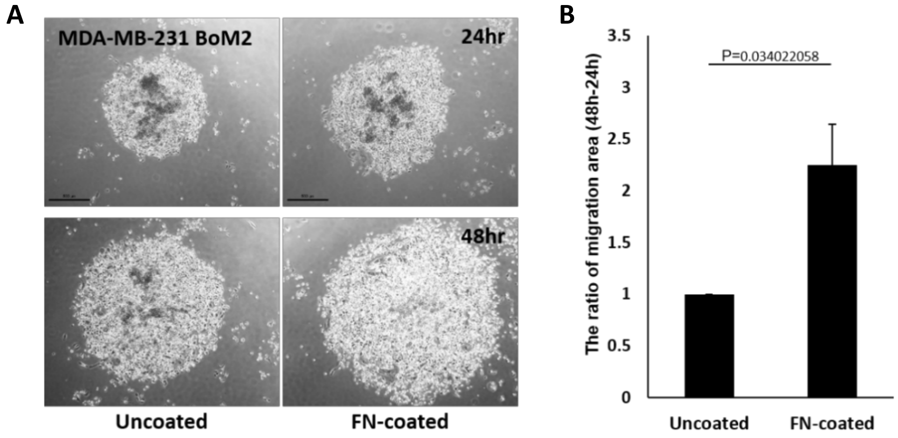

Supplementary Figure 6. The effect of fibronectin on migration of BoM2 cells derived from 3D aggregates.

BoM2 cell aggregated formed in ultra-low attachment round bottom plates for 48hours were transferred on uncoated or fibronectin Ⅳ-coated culture glassed and the areas covered with migrated cells from the aggregated were measured and compared. (means ± s.e.m, Student’s t-test)

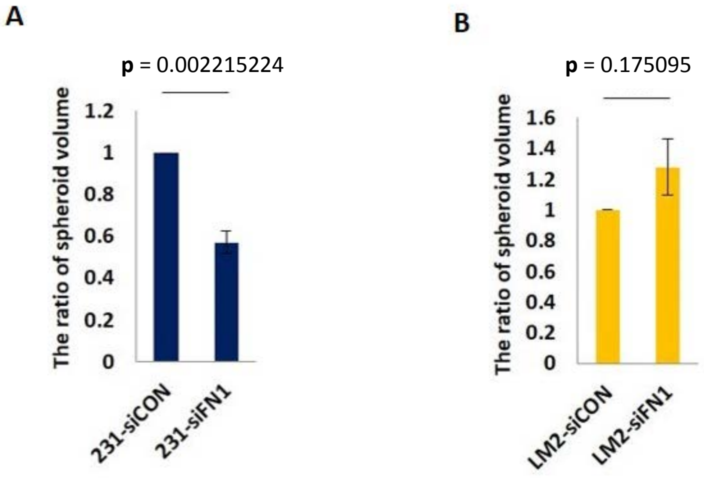

Supplementary Figure 7. The rate of cell spheroids volume regulated by fibronectin

FN1 or control siRNA transfected MDA-MB-231 parental and LM2 cells (5,000cells/well) were cultured in ultra-low attachment 96-well round bottom plates to form spheroid for 48hours and the volume of spheroid at 48hr were measured by Image J (A and B). (means ± s.e.m, Student's t-test)

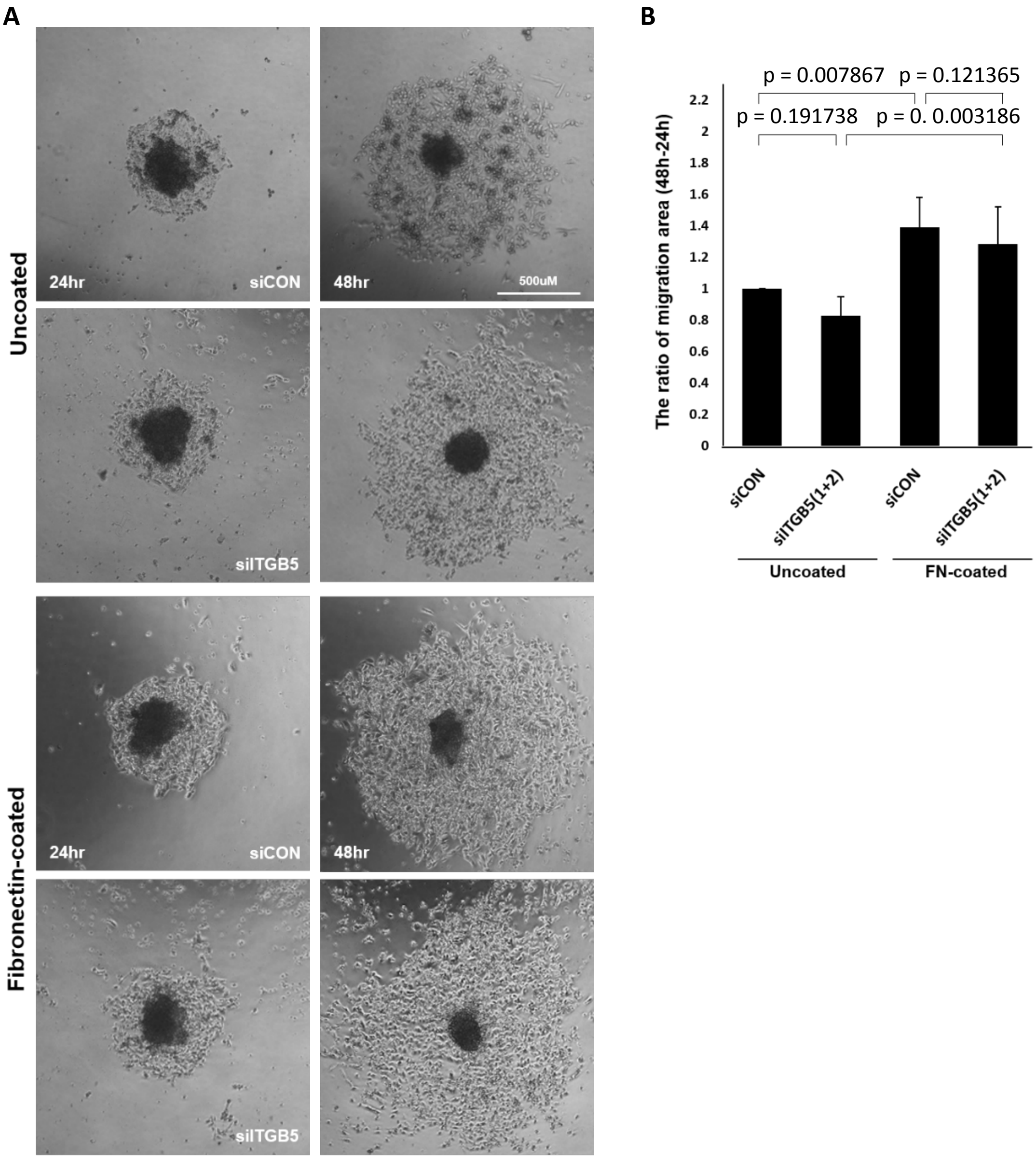

Supplementary Figure 8. The effect of ITGB5 silencing on cell migration from 3-dimensional cellular spheroids

ITGB5 or control siRNA transfected MDA-MB-231 parental cells were cultured in ultra-low attachment 96-well round bottom plates to form spheroid for 48hours and the cell spheroids were re-plated on uncoated or fibronectin-coated 2D culture plates. (A) Cells migrated from the spheroids and the area covered by cellular monolayers were measured at 24hour point and 48hour point by using Image J, and (B) the area changes from 24hour point to 48hour point were calculated and compared. (means ± s.e.m, Student's t-test)

Figure 1A and S. Figure 1A

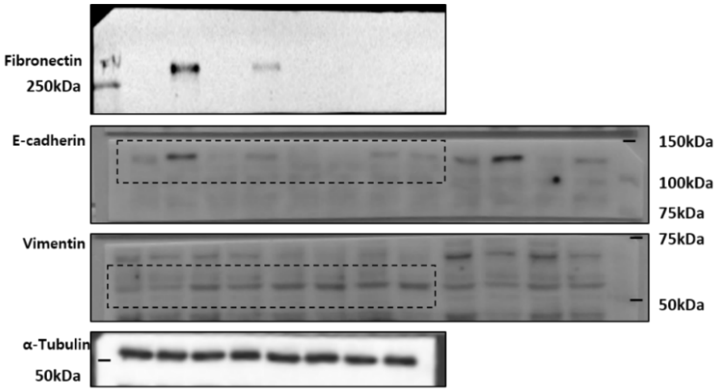

S. Figure 1B

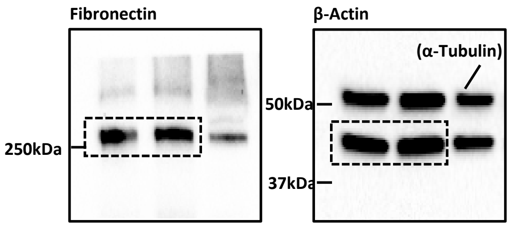

Figure 1B

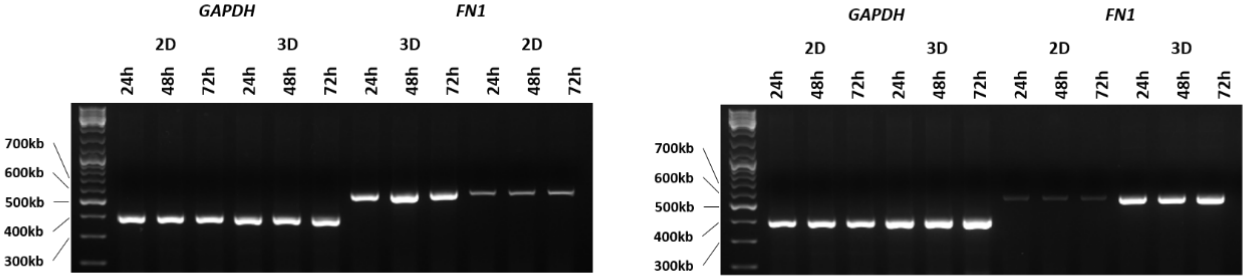

Figure 1C-1

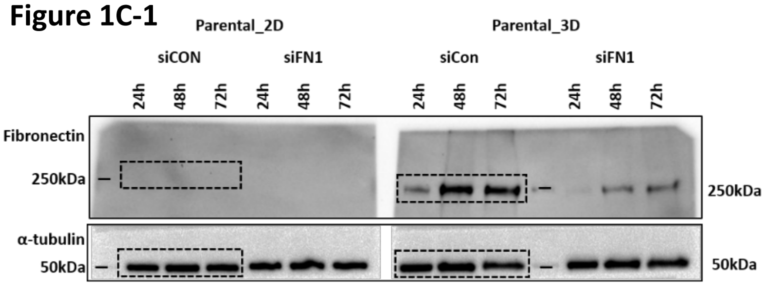

Figure 1C-2

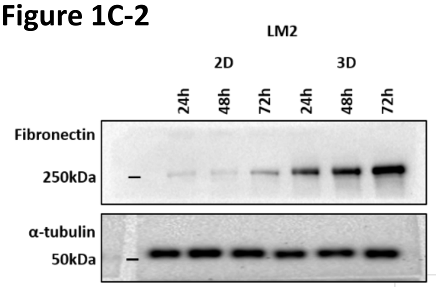

Figure 1D

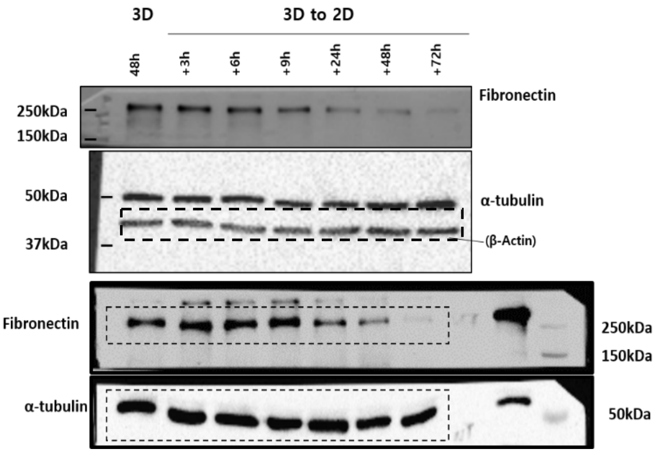

Figure 1E

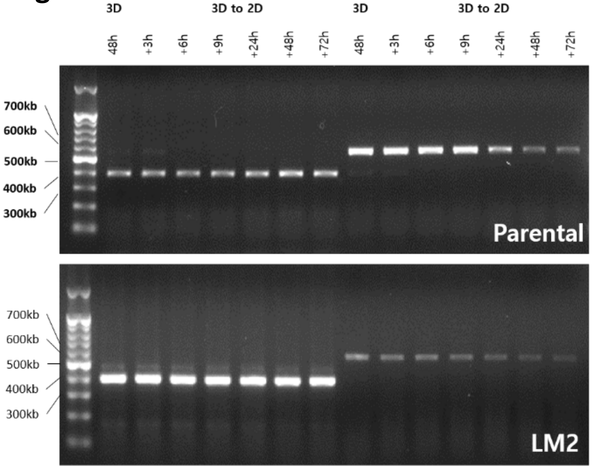

Supplemental Figure 9. Uncropped immunoblots (A, C, and D) and electrophoretic gels (B and E) for panels in Figure 1 and supplementary figure 1.

Figure 2A

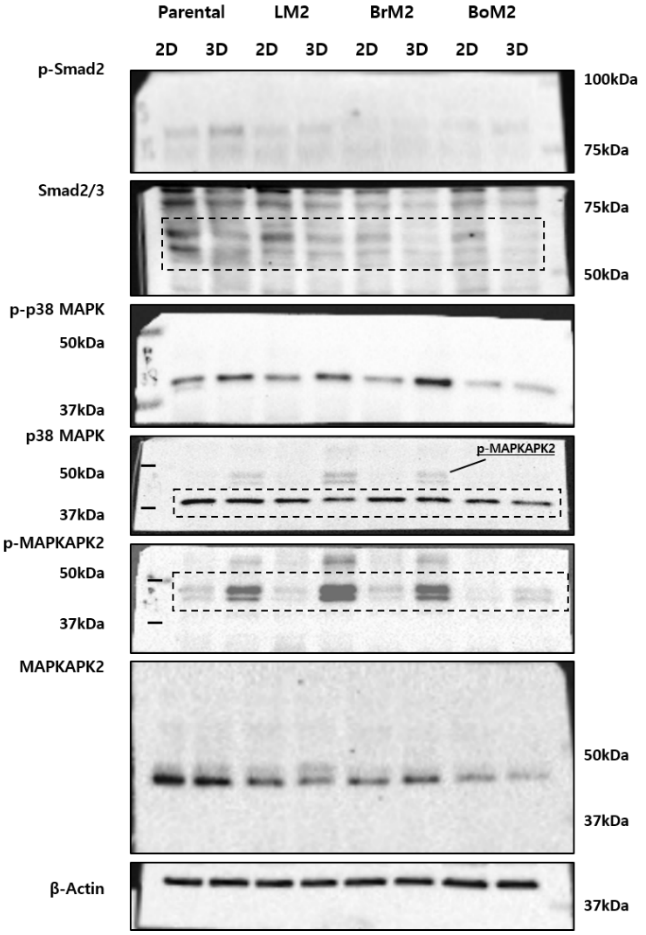

Figure 2B

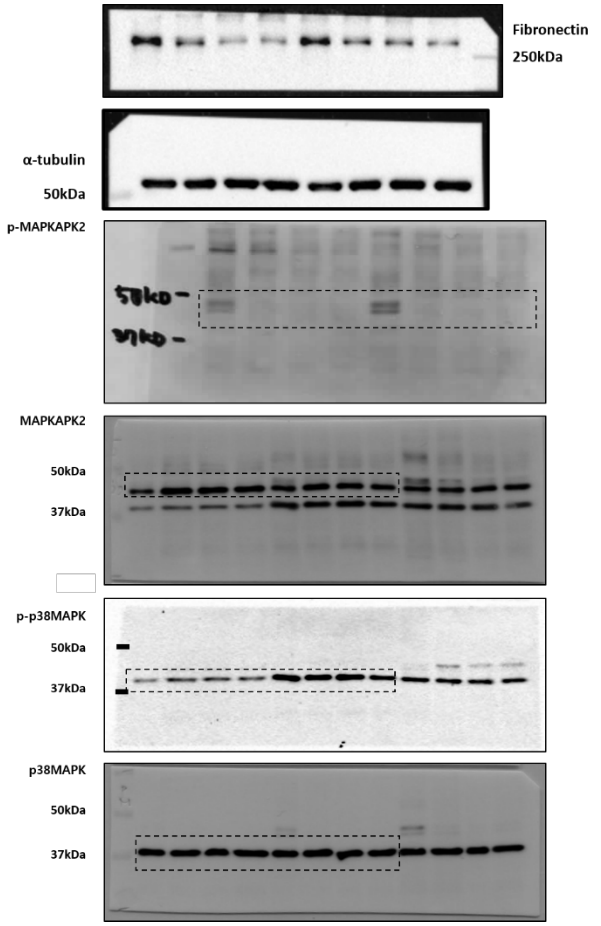

Supplemental Figure 10. Uncropped immunoblots (A and B) for panels in Figure 2.

Figure 3A

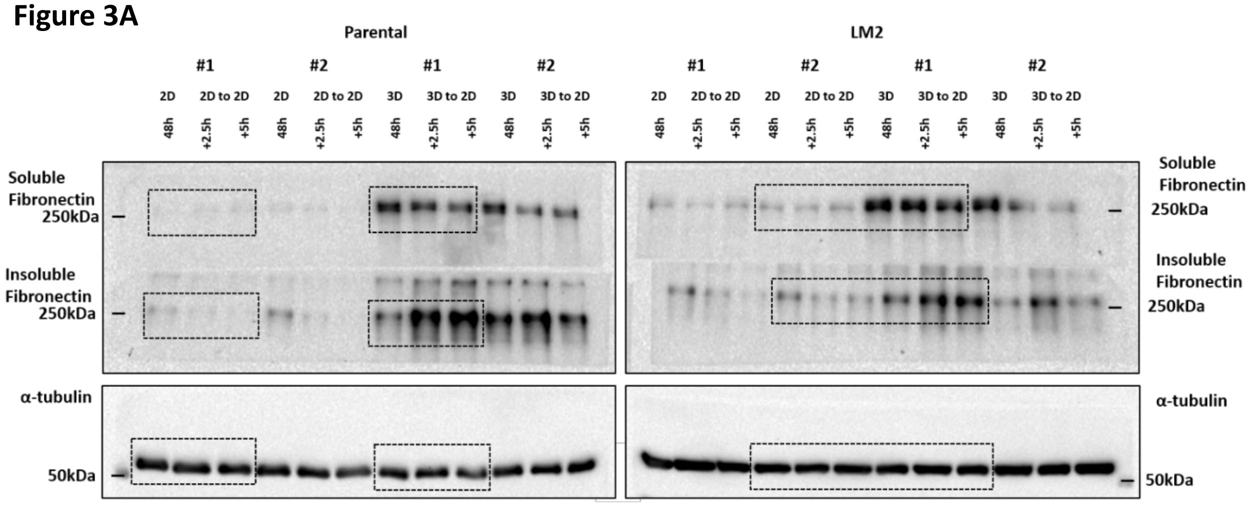

Figure 3C

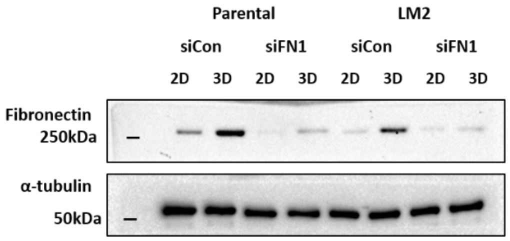

Supplemental Figure 11. Uncropped immunoblots (A and C) for panels in Figure3.

Figure 5C

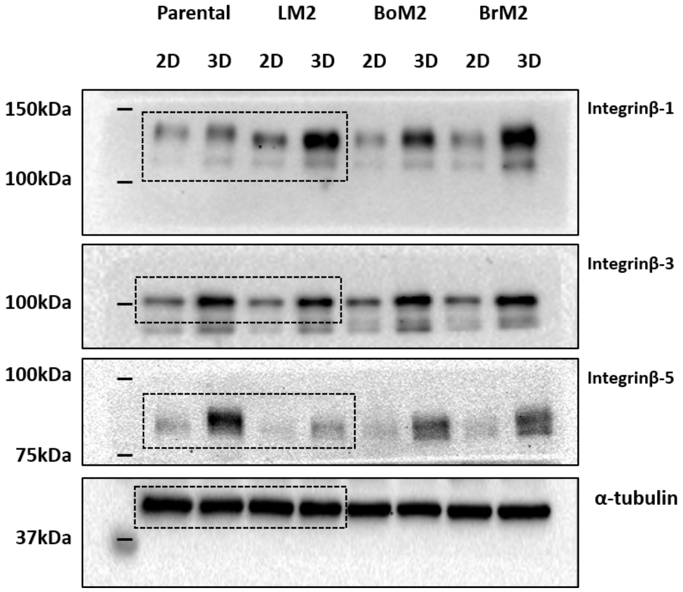

Figure 5D

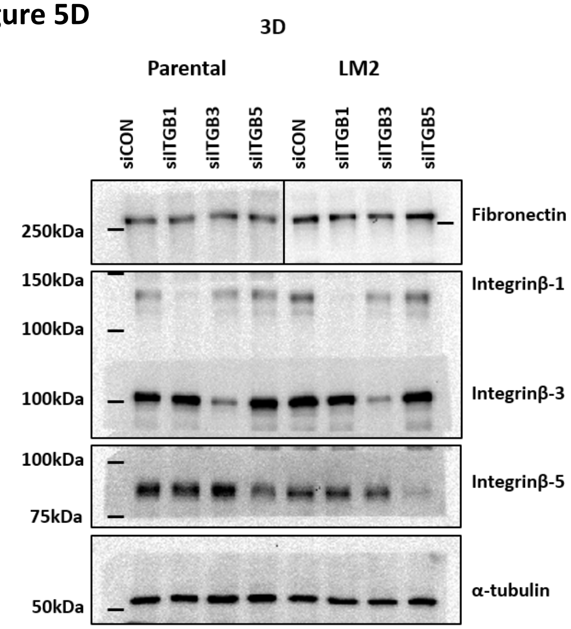

Figure 5G

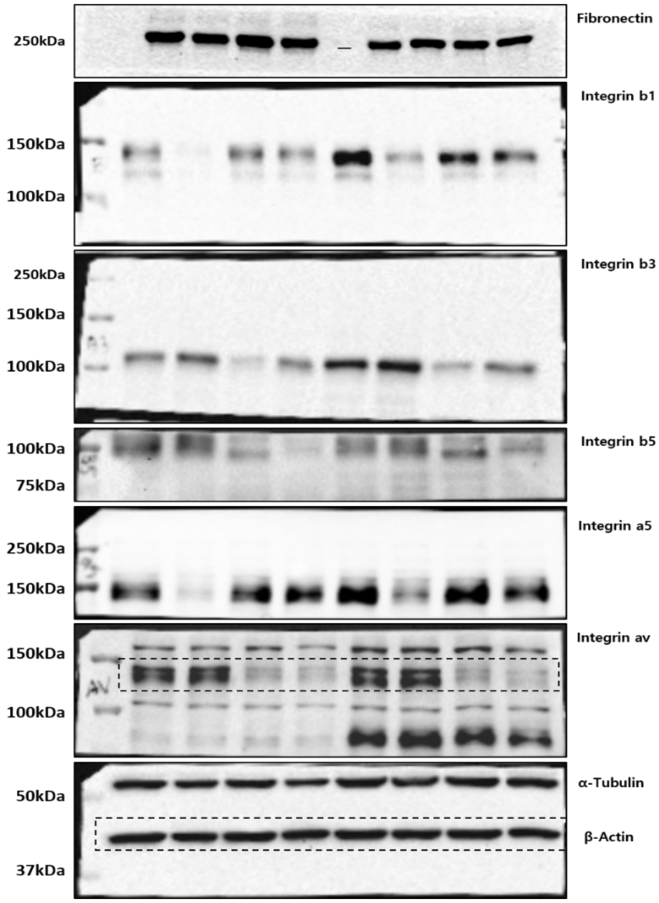

Supplemental Figure 12. Uncropped immunoblots (C, D, and G) for panels in Figure 5.

Figure 6A

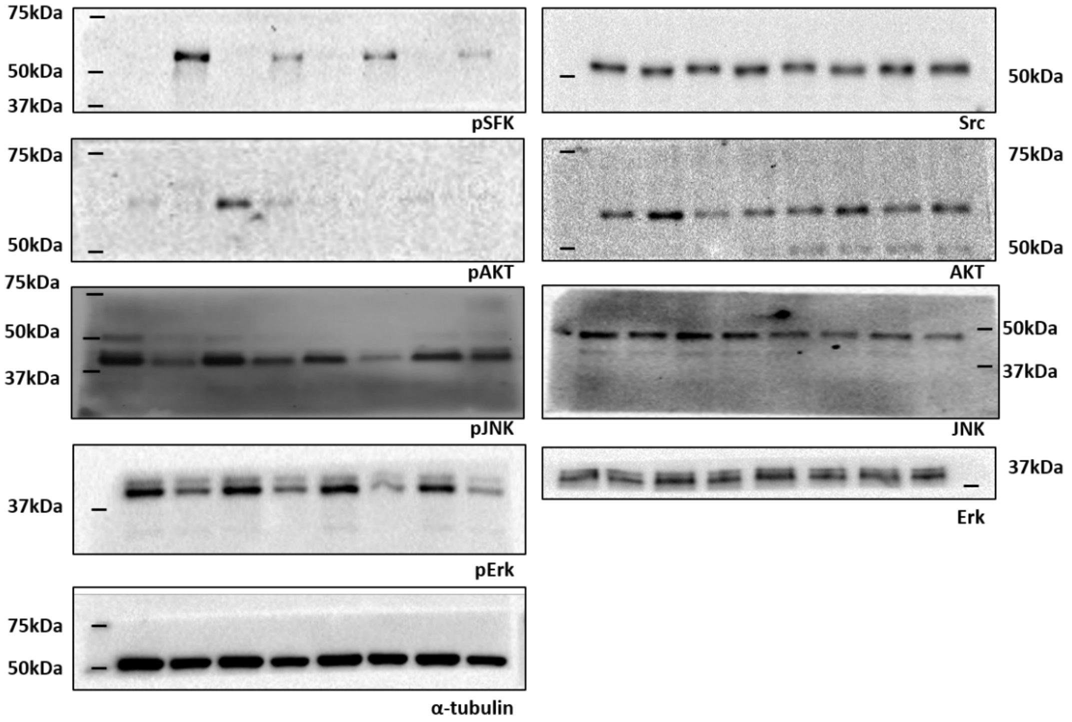

Figure 6B

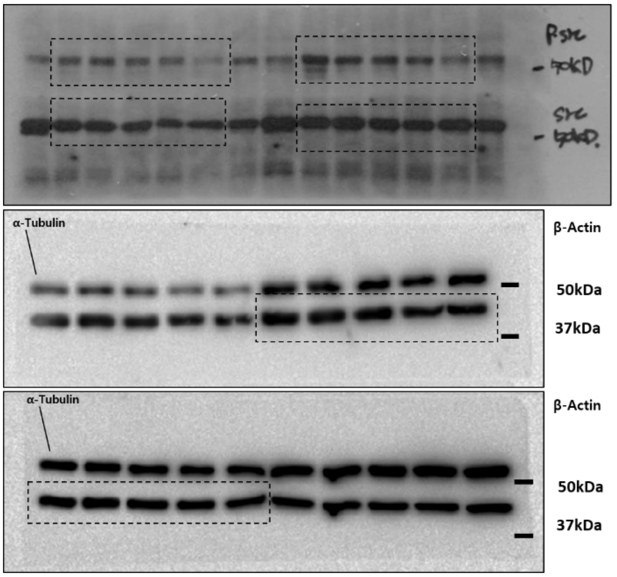

Supplemental Figure 13. Uncropped immunoblots (A and B) for panels in Figure 6.
